# Neurodegenerative NMNAT2 Deficiency Promotes APP Processing in a SARM1-Dependent Manner

**DOI:** 10.3390/cells15121100

**Published:** 2026-06-17

**Authors:** Andrea Enriquez, Sen Yang, Karen Ling, Paymaan Jafar-Nejad, Hui-Chen Lu

**Affiliations:** 1 Gill Institute for Neuroscience, Indiana University, Bloomington, IN 47405, USA; anenri@iu.edu (A.E.);; 2 Program in Neuroscience, Indiana University, Bloomington, IN 47405, USA; 3 Department of Psychological and Brain Sciences, Indiana University, Bloomington, IN 47405, USA; 4 Neuroscience Drug Discovery, IONIS Pharmaceuticals Inc., Carlsbad, CA 92010, USA

**Keywords:** proteostasis, NMNAT2, SARM1, NAD^+^ metabolism, APP processing

## Abstract

**Highlights:**

**What are the main findings?**

- Reduced glucose metabolism results in APP processing deficits and APP-CTF accumulation in cortical neurons.
- NMNAT2 loss and SARM1 activation trigger early glycolytic failure followed by a later mitochondrial decline, driving progressive defects in APP processing and proteostasis.

**What are the implications of the main findings?**

- The NMNAT2–SARM1 axis links energy homeostasis to APP processing and proteostasis.
- SARM1 knockdown offers a therapeutic strategy beyond NAD^+^ supplementation to address metabolic deficits and their downstream effects on proteostasis.

**Abstract:**

Metabolic dysfunction and proteinopathy are hallmarks of neurodegenerative disease, yet their mechanistic interplay remains poorly understood. Here, we show that loss of the neuronal NAD^+^-synthesizing enzyme Nicotinamide mononucleotide adenylyltransferase 2 (NMNAT2) disrupts amyloid precursor protein (APP) processing in cortical neurons, leading to accumulation of APP C-terminal fragments (APP-CTFs). NMNAT2 deficiency lowers the NAD^+^/NADH redox ratio coincident with APP-CTF buildup. Temporal profiling reveals a biphasic increase in APP-CTFs, with an initial gradual rise followed by rapid accumulation, paralleling the expansion of differentially expressed proteins. Pathway analysis indicates early activation of JNK/MAPK signaling, followed by late-stage suppression of mitochondrial pathways and induction of endoplasmic reticulum stress and unfolded protein response programs. Seahorse analyses reveal early glycolytic impairment followed by deficits in mitochondrial respiration. Knockdown of the NAD^+^ hydrolase sterile alpha and TIR motif-containing protein 1 (SARM1) restores mitochondrial function and normalizes APP-CTF levels in NMNAT2 knockout neurons, whereas NAD^+^ supplementation provides only modest rescue. Together, these data demonstrate that neuronal NAD^+^ depletion drives progressive, SARM1-dependent disruption of glucose metabolism and proteostasis, impairing APP processing. The NMNAT2–SARM1 axis thus links metabolic stress to proteinopathy and highlights SARM1 as a central mediator of neurodegenerative dysfunction.

## 1. Introduction

Neurodegenerative diseases involve system-level dysfunctions that are caused by a variety of environmental and genetic factors. FDG-PET imaging studies have revealed progressive cerebral glucose hypometabolism in patients with Alzheimer’s disease (AD), Parkinson’s disease (PD), and Huntington’s disease (HD) [1,2,3,4]. Their pathological features include progressive, age-dependent proteinopathy and neuronal loss [5,6,7], and growing evidence suggests that aging-associated declines in cellular energy metabolism contribute to the onset and progression of neurodegeneration [8,9,10,11]. Amyloid β (Aβ) deposition studies in AD patients have shown that brain regions with Aβ plaques also exhibit significant hypometabolism of glucose [12]. Currently, the mechanistic link between glucose hypometabolism and proteinopathy remains elusive.

Nicotinamide adenine dinucleotide (NAD^+^) occupies a central position at the interface of energy metabolism [13], redox balance [14], and pathways involved in signaling cascades that regulate proteostasis [15]. Protein processing, which includes protein folding, post-translational modifications, trafficking through organelles, and proteasomal clearance, involves a wide array of molecular mechanisms and organelles and is particularly sensitive to metabolic stress [16,17,18,19]. Nicotinamide mononucleotide adenylyl transferase 2 (NMNAT2) is the most abundant NAD^+^-synthesizing enzyme in mammalian cortical neurons [20,21,22]. Its expression is reduced across various proteinopathies, including AD, PD, HD [23], as well as in the spinal cord of amyotrophic lateral sclerosis patients [24]. Our recent studies show that deleting NMNAT2 from cortical glutamatergic neurons in mice results in defective glucose metabolism and several neurodegenerative phenotypes, including axonal amyloid precursor protein (APP) accumulations and neurodegeneration in a sterile alpha and TIR motif-containing protein 1 (SARM1)-dependent manner [25,26]. SARM1 is an NAD^+^ hydrolase that is activated when NMNAT2 levels are reduced [27], leading to NAD^+^ depletion and axonal degeneration [28].

APP is a transmembrane protein that is primarily cleaved by two canonical pathways: non-amyloidogenic APP processing or amyloidogenic APP processing, both of which are driven by α-, β-, and γ-secretases [29]. The cleavage by α- and β-secretases generates APP C-terminal fragments (APP-CTFs), and subsequent cleavage by γ-secretases leads to the production of Aβ. Significant work has focused on abnormal APP cleavage for neurotoxic Aβ generation [30]. However, there is growing evidence highlighting the toxic effects of APP-CTFs, including decreased mitochondrial respiration, endolysosomal dysfunction, synapse loss, neuronal hyperexcitability, and mitophagy [31,32,33,34,35,36]. Bretou et al. [37] showed that both α- and β-derived CTFs reproduce endolysosomal dysfunction, indicating that toxicity derives from APP-CTF signaling rather than from a specific cleavage product. Additionally, intracellular accumulation of β-CTFs in human neurons directly impairs lysosomal function [38]. Studies with AD mouse models show that β-CTF accumulation directly contributes to synaptic dysfunction and non-cognitive behavioral alterations [36]. Interestingly, CTFs could be released with exosomes upon neuronal lysosomal dysfunction [39]. The proteolytic processing of APP can be altered by disrupted intracellular trafficking, since secretases are localized in different cellular compartments, and changes in APP trafficking can affect its access to these enzymes [40,41,42]. Moreover, defective energy homeostasis has also been shown to alter APP processing [43,44,45].

We have shown that neuronal NAD^+^ deficiency caused by NMNAT2 deletion leads to axonal APP accumulation and axon degeneration in a SARM1-dependent manner [25]. In our previous studies, APP aggregates in NMNAT2 knockout (KO) axons were detected using an antibody against the C-terminal APP epitope. Given the known influence that energy metabolism has on APP processing and the detrimental effects of elevated APP-CTFs on neuronal health, we hypothesized that NAD^+^ deficiency disrupts APP processing, thereby increasing APP-CTFs. To determine the role of the NMNAT2–SARM1 axis in APP processing, we used a combination of biochemical, metabolic, proteomic, pharmacological, and antisense oligonucleotide-based approaches. Our results show that NMNAT2 deficiency induces mechanistically distinct yet interconnected proteostatic and metabolic impairments, leading to a progressive accumulation of APP-CTFs. Furthermore, we identify the NMNAT2–SARM1 axis as a critical regulator of APP processing in cortical neurons.

## 2. Materials and Methods

### 2.1. Mice

Blad mice with an NMNAT2 null mutation were generated by transposon-mediated gene-trap mutagenesis as previously described [46]. Both male and female mice were used throughout this study. All mice were housed under standard conditions with ad libitum food and water and maintained on a 12 h light/dark cycle. Animal care and experimental procedures were carried out in compliance with the NIH Guidelines for the Care and Use of Laboratory Animals and approved by the Institutional Animal Care and Use Committees at Indiana University.

### 2.2. Genotyping

Genotyping lysates were prepared by placing embryonic tail samples in digestion buffer (50 mM KCl, 10 mM Tris-HCl, 0.1% Triton X-100, 0.2 mg/mL proteinase K, pH 9.0) and incubating at 60 °C for 15 min in a thermal shaker set to 1500 rpm. Samples were then incubated at 95 °C for 10 min to denature the proteinase K and centrifuged at max speed in a microcentrifuge for 5 min. The supernatants were used as DNA templates for polymerase chain reactions using the EconoTaq^®^ Plus Green 2X (Biosearch™ Technologies, Guildford, UK) master mix or the QIAGEN^®^ Fast Cycling PCR Kit (QIAGEN, Germantown, MD, USA). Samples were loaded onto a 1.6% agarose gel and ran at 135 V for 30 min. The primer sequences used for genotyping have been previously described [25].

### 2.3. Primary Cortical Neuron Culture

Primary cortical mouse neurons were prepared using E14.5–16.5 embryos from heterozygous (het) NMNAT2-Blad mouse matings. Cortices of both male and female embryos were dissected and genotyped, to then be sorted into wild-type (WT) and KO groups. Following dissection and genotyping, the pooled tissue was prepared for plating using the Papain Dissociation System (Worthington^®^, Lakewood, NJ, USA) following the manufacturer’s protocol. Cells were counted using a hemocytometer and plated at a density of 1.43 × 10^5^ cells/cm^2^. Primary neurons were maintained in Neurobasal Media (Gibco™, Waltham, MA, USA) supplemented with B-27 (Gibco™), GlutaMAX™ (Gibco™), and penicillin-streptomycin (Gibco™). All cell culture preparations were performed under sterile conditions, and cells were incubated at 37 °C with 5% CO_2_ and appropriate humidity. One third of the media was replenished every 3 days. Samples were collected at day-in-vitro (DIV) indicated in Section 3.

### 2.4. Western Blotting

Neurons were lysed using 150 μL of RIPA buffer per well [20 mM of Tris-base, 150 mM of NaCl, 2 mM EDTA, 1% Triton X-100, 0.5% sodium deoxycholate, 0.1% sodium dodecyl sulfate (SDS), 1× cOmplete™ Mini protease inhibitor cocktail (Roche^®^, Basel, Switzerland), and phosphatase inhibitor cocktail 3 (Sigma-Aldrich^®^ P0044, St. Louis, MO, USA), pH 7.4] and homogenized for 15 s with a pestle motor using disposable pellet pestles. Samples were then sonicated using a Branson Sonifier^®^ 250 (Emerson, St. Louis, MO, USA) for 10 intervals set at duty cycle 30/output 3 and stored at −80 °C.

Protein concentration was measured using the Pierce™ BCA Protein Assay Kit (Thermo Scientific™, Waltham, MA, USA) and samples were prepared for loading in Laemmli SDS buffer (Thermo Scientific™). Samples were normalized to 4–20 μg of protein per lane and loaded onto a 16.5% SDS-polyacrylamide gel. Gels were run at 80 V for 20 min, then at 110 V until the samples reached the bottom of the gel. Protein was then transferred to a nitrocellulose membrane with a 0.22 μm pore size using a tank transfer system (Bio-Rad^®^, Hercules, CA, USA). For sample normalization, nitrocellulose membranes were stained with Ponceau S for 30 s, rinsed 3× with Milli-Q^®^ dH_2_O (Sigma-Aldrich^®^), and imaged on a Gel Doc™ XR+ imager (Bio-Rad^®^). Membranes were blocked for 1 h at room temperature with blocking solution (1:1 BlockOut^®^ Universal Blocking Buffer (Rockland Immunochemicals, Pottstown, PA, USA) and 0.1% TBST), then incubated for 24 h with APP primary antibody (ab32136, Abcam^®^, Cambridge, UK) diluted in blocking solution. The next day, membranes were washed with 0.1% TBST (3 × 10 min) and incubated for 2 h with the secondary antibody IRDye 680 LT (92-68021, LI-COR^®^, Lincoln, NE, USA) diluted in blocking solution. Membranes were then washed with 0.1% TBST (3 × 10 min) and stored in TBS until imaging. Western blot images were acquired with an Odyssey^®^ scanner (LI-COR^®^) and software, and protein abundance was quantified using the NIH Fiji ImageJ (Version 2.16.0/1.54i) [47]. Ponceau S staining was used to normalize the signal in each sample. Briefly, each lane was quantified using Fiji’s Gels function. A box of equal size was drawn over each sample lane and over a background area, and signal intensity from each box was plotted using Fiji’s Plot Lanes function. The area under the curve was measured, and each sample’s total protein amount was calculated by subtracting background signal from the total signal. For APP analysis, two approaches were used to quantify signal. Total expression band values were obtained using the Fiji Gels built-in analysis function. Briefly, boxes of the same size were placed over each band, and lane intensity was plotted using Fiji’s Plot Lanes function. Area under the curve was then extracted from each plot, and intensity was normalized to WT controls in each gel. For the intensity signal curve plots, all gel sizes were matched by resizing the distance between the 25 and 10 kilodalton (kDa) molecular weight markers to 200 pixels using the Resize function. Once gels were resized, a line of equal length was placed at the 20 kDa protein marker and positioned at the center of the bands signal for each sample. The Fiji Plot Profile function was used to create response curves of signal intensity at each point of the line, and signal across this set distance was acquired for each sample. Signal intensity was normalized to the WT controls in each gel.

### 2.5. BACE1 Inhibitor Treatment

β-Secretase Inhibitor IV (Cayman Chemical, Ann Arbor, MI, USA, 23388), a known inhibitor for β-site amyloid protein cleaving enzyme 1 (BACE1), was dissolved in DMSO to a stock concentration of 1 mM and stored at −20 °C. For experiments, the inhibitor was diluted in cell culture medium to a working concentration of 3 µM. At DIV10, cells were treated with either DMSO or a BACE1 inhibitor (B-Inh) by replacing one-third of the medium in each well, resulting in a final concentration of 1 µM. Cells were collected at DIV13 for Western blotting.

### 2.6. NAD^+^ Supplementation

NAD^+^ (Roche^®^, NAD100-RO) was dissolved at a stock concentration of 100 mM in 1× PBS, filtered with a 0.22 µM syringe filter, and stored at −80 °C for up to 7 days. On the first day of supplementation, NAD^+^ was added to the culture medium at a final concentration of 1 mM. The same volume of PBS was added to the Neurobasal medium to use as a control. In the following days, one-third of the culture medium was refreshed daily with 1 mM NAD^+^ or PBS until cells were collected.

### 2.7. Antisense Oligonucleotide Treatment

Antisense oligonucleotides (ASOs) used in this study have been previously described and validated [25]. A non-targeting ASO, 5′-CCTATAGGACTATCCAGGAA-3′ (Ctrl ASO) was used as a control. An ASO targeting mouse SARM1 mRNA, 5′-GGTAAGAGCCTTAGGCACGC-3′ (SARM1 ASO) was used to knockdown (KD) SARM1 expression. To start treatment, ASOs were diluted in neuronal culture media and added to neurons at a final concentration of 5 µM, and one third of the culture medium was refreshed every 3 days with 5 μM concentration of ASOs.

### 2.8. NAD^+^/NADH-Glo™ Bioluminescent Assay

NAD^+^/NADH measurements were carried out as previously described [25]. Briefly, WT and KO neurons were cultured in 96-well plates at a density of 1.3 × 10^3^ cells/mm^2^. Following Promega’s NAD^+^/NADH-Glo™ Assay protocol (Promega^®^ G9071, Madison, WI, USA), DIV8 or DIV12 neurons were lysed in DTAB base buffer (100 mM Sodium Carbonate, 20 mM Sodium Bicarbonate, 10 mM Nicotinamide, 0.05% TritonX-100, pH 10–11). Lysates were then split for total NAD^+^ + NADH and NADH-only measurements, with selective degradation of NAD^+^ in the NADH samples prior to detection. The Promega detection reagent was then added to all wells, and the enzymatic cycling reaction that generates a luminescent signal proportional to NADH content was measured with a CLARIOstar^®^ plate reader (BMG LABTECH, Cary, NC, USA). Luminescence was measured using a microplate reader, and NAD^+^ levels were calculated by subtracting NADH from total values. NAD^+^ and NADH measurements were normalized to protein concentration measured with Pierce™ BCA Protein Assay Kit (Thermo Scientific™).

### 2.9. Seahorse XF Glycolytic Rate Assay

Cellular glycolysis was measured using the Seahorse XF Glycolytic Rate Assay Kit (Agilent^®^, Santa Clara, CA, USA, 103344-100) following the manufacturer’s protocol. Primary cortical neurons were prepared as described above. Neurons were seeded in Seahorse XF24 Cell Culture Microplates at a density of 40,000 cells per well in 250 μL of XF DMEM medium (Agilent^®^, 103575-100). Assays were performed on an XFe24 Seahorse Analyzer (Agilent^®^) at DIV8 and DIV12. Following basal measurements, the medium is injected with 15 µM N-methyl-D-aspartic acid (NMDA) and 2 µM Glycine. Cells are sequentially treated with the mitochondrial inhibitor rotenone/antimycin A (Rot/AA) at 0.5 µM, and glycolysis inhibitor 2-deoxyglucose (2DG) at 50 mM. Extracellular acidification rate (ECAR) and oxygen consumption rate (OCR) are recorded and used to calculate glycolytic proton efflux rate (glycoPER). GlycoPER was analyzed using Wave software (Agilent^®^, version 2.6.4) following the manufacturer’s instructions. Data were normalized to total protein content per well using the Pierce™ BCA Protein Assay Kit (Thermo Scientific™).

### 2.10. Seahorse XF Cell MitoStress Test

Mitochondrial function was measured using the Seahorse XF Cell MitoStress Test Kit (Agilent^®^, 103015-100) following the manufacturer’s protocol. Primary cortical neurons were prepared as described above. Neurons were seeded in Seahorse XF24 Cell Culture Microplates at a density of 40,000 cells per well in 250 μL of XF DMEM medium (Agilent^®^, 103575-100). Assays were performed at DIV8 and DIV12 using an XFe24 Seahorse Analyzer (Agilent^®^). Sequential injections of mitochondrial modulators were performed at the following final concentrations: 1.5 µM oligomycin, 2 µM carbonyl cyanide-4 (trifluoromethoxy) phenylhydrazone (FCCP), and 0.5 µM Rot/AA. OCR was recorded and analyzed using Wave software (Agilent^®^) following the manufacturer’s instructions. Data were normalized to total protein content per well using the Pierce™ BCA Protein Assay Kit (Thermo Scientific™).

### 2.11. Proteomic Profiling

WT control and KO neurons were plated on 12-well plates as described above. At DIV8 or 12, cells were rinsed 3× with ice-cold PBS. To each well were added 300 μL of PBS and cells were detached from the plate using a Falcon^®^ sterile cell scraper (Corning^®^, Corning, NY, USA). Two wells were combined into 1 sample and centrifuged at 300× *g* for 8 min. Cell pellets were rinsed once with ice-cold PBS and centrifuged at 300× *g* for 6 min. Samples were flash frozen in liquid nitrogen and stored at −80 °C.

Liquid chromatography-mass spectrometry (LC-MS) experiments, along with preliminary data normalization and analysis, were conducted at the Center for Proteome Analysis at Indiana University School of Medicine (Indianapolis, IN, USA). Details on mass spectrometry methodology and data analysis can be found in the Supplementary Data.

Gene set enrichment analysis was performed using Enrichr https://maayanlab.cloud/Enrichr/ (accessed on 29 January 2024) and STRING https://string-db.org (accesses on 20 August 2024). Proteins with significant abundance ratio *p*-values were separated into upregulated and downregulated groups. Protein lists were imported into Enrichr and Gene Ontology (GO) Biological Processes (BP) were extracted. Data lists were also uploaded onto STRING to create full confidence string networks using databases, experiments, and co-expression as active interaction sources, with a minimum required interaction score of 0.4. Clustering was done by using the DBSCAN method with an epsilon parameter of 4, and top GO terms for BP, molecular function (MF), and cellular compartment (CC) terms were extracted by enrichment analysis.

### 2.12. Statistical Analyses

All data sets were tested for normality of residuals using the D’Agostino–Pearson normality test. For data sets with normally distributed residuals, we used one-way ANOVA with Tukey’s multiple comparison test, two-way ANOVA with Tukey’s multiple comparison test, or unpaired student’s *t*-test. For data sets that did not pass the D’Agostino–Pearson normality test, one-way ANOVA was replaced by Kruskal–Wallis test and Dunn’s multiple comparison test; two-way ANOVA was replaced by Multiple Mann–Whitney test or re-formatted for Kruskal–Wallis test; unpaired student’s *t*-test was replaced by the Mann–Whitney test. Correlation plots were analyzed using simple linear regression and the slope was tested against zero using *t*-test. The criterion for statistical significance was set at *p*  <  0.05 for all statistical analyses.

## 3. Results

### 3.1. Neuronal NMNAT2 Loss Results in SARM1-Dependent APP-CTF Accumulations

**Figure 1:**
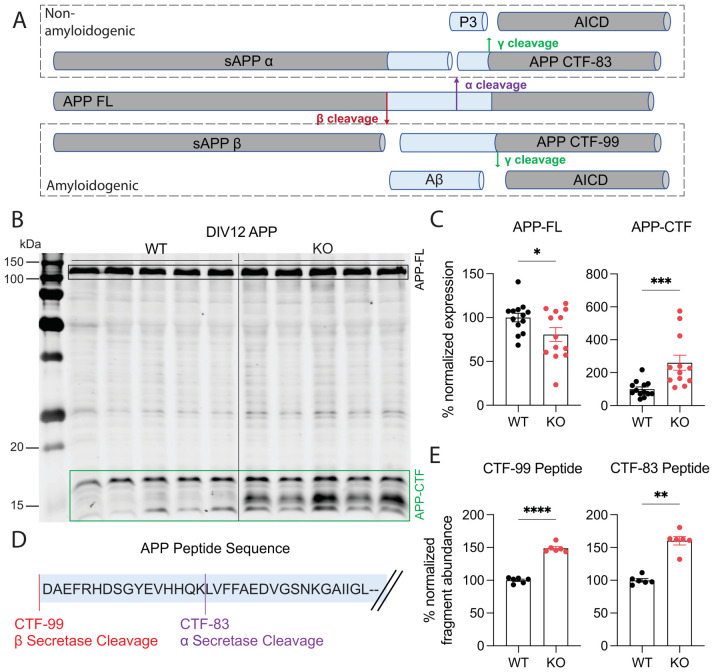
APP processing alterations in NMNAT2 KO neurons. (**A**) Schematic diagram of non-amyloidogenic and amyloidogenic APP processing pathways. (**B**) Western blot of lysates from DIV12 wild-type (WT) and NMNAT2 knockout (KO) primary mouse cortical neuron cultures show APP full-length (APP-FL) and APP C-terminal fragments (APP-CTF). (**C**) Western blot quantification analysis. Intensity of APP-FL band and APP-CTF cluster of bands relative to WT neurons. DIV12; APP-FL *n* = 13 WT, 13 KO; APP-CTF n = 13 WT, 12 KO. APP-FL data analyzed with unpaired *t*-test and APP-CTF data analyzed with Mann–Whitney test. *, *p* = 0.049, ***, *p* = 0.0002. Samples were collected from three independent experiments. (**D**) Peptide sequence of APP illustrating the location at which beta and alpha secretases cleave the protein. (**E**) LC-MS/MS quantification of peptide sequences for the CTF-99 fragment as a result of beta-secretase cleavage and CTF-83 fragment as a result of alpha-secretase cleavage. DIV12; n = 6 WT, 6 KO; CTF-99 data was analyzed with unpaired *t*-test and CTF-83 data analyzed with Mann–Whitney test. **, *p* = 0.0022, ****, *p* < 0.0001. Samples were collected from two independent experiments. All bar graphs represent mean ± SEM.

The cleavage of full-length APP (APP-FL) by an α-secretase yields a secreted-APP α (sAPP-α) fragment and a C-terminal fragment 83 (CTF-83) [29] (Figure 1A). The subsequent cleavage of CTF-83 by γ-secretase results in P3 and the APP Intracellular Domain (AICD). When APP-FL gets cleaved by β-secretase, it produces a secreted-APP β (sAPP β) fragment and a C-terminal fragment 99 (CTF-99). CTF-99 is then cleaved by γ-secretase into an Aβ peptide and an AICD fragment. To evaluate the effects of NMNAT2 loss on APP processing, we first performed Western blotting analyses to quantify APP-FL and APP-CTFs in DIV12 primary control and NMNAT2 KO neurons using a C-terminal targeting APP antibody (Figure 1B). We found that NMNAT2 KO neurons exhibit 2-fold higher expression of APP-CTFs, ranging from 10–18 kDa, and ~20% less APP-FL than WT neurons (Figure 1C). The cleaving sites of APP processing secretases only differ by a few amino acids [29], making it challenging to clearly separate fragment products by size in Western blots. Thus, we conducted LC-MS proteomics on DIV12 WT and NMNAT2 KO samples to determine the abundance of CTF-83 and CTF-99 by searching peptide sequences that match the α- and β-secretase cleavage sites. These analyses found that both CTF-83 and CTF-99 corresponding peptides were significantly increased in DIV12 NMNAT2 KO neurons (Figure 1E).

To further confirm the increase of CTF-83 and CTF-99, we treated WT and NMNAT2 KO neurons with 1 µM β-Secretase Inhibitor IV, a well-characterized BACE1 inhibitor [48,49], from DIV10–13 to examine whether the band corresponding to CTF-99 is decreased in DIV13 NMNAT2 KO neurons (Supplementary Figure S1A). BACE1 inhibition increased APP-FL in both WT and KO-treated samples (Supplementary Figure S1B), confirming that BACE1 is active in DIV10–13 cortical neurons and that the inhibitor is effective. The shift of CTF distributions to lower molecular weights upon BACE1 inhibition in KO neurons allowed us to confirm the locations of CTF-99 and CTF83. However, BACE1 inhibition did not reduce the overall APP-CTF levels in KO neurons when compared to the vehicle-KO group (Supplementary Figure S1C). No significant differences in either CTF-99 or CTF-83 levels were found between vehicle-treated and BACE1-inhibited WT or KO groups (Supplementary Figure S1D,E). These findings suggest that BACE1 is not a major contributor to the elevated APP-CTF levels upon NMNAT2 loss.

**Figure 2:**
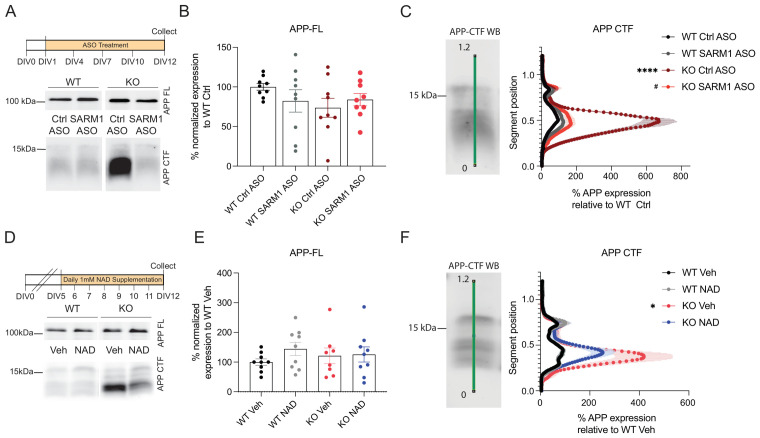
SARM1 KD resolves APP processing phenotypes, while NAD^+^ supplementation partially rescues them. (**A**) Western blot of APP-FL and CTF in WT and KO neurons treated with Ctrl ASO or SARM1 targeting ASO. (**B**) Quantification of APP-FL band expression relative to WT Ctrl ASO. Data was analyzed using one-way ANOVA with Tukey’s multiple comparisons test. (**C**) Quantification of band intensity across APP-CTFs. Data was analyzed using Kruskal–Wallis test with Dunn’s multiple comparisons test. WT Ctrl ASO vs. KO Ctrl ASO ****, *p* < 0.0001. KO Ctrl ASO vs. KO SARM1 ASO #, *p* = 0.0197. *n* = 9 WT Ctrl ASO, 9 WT SARM1 ASO, 9 KO Ctrl ASO, 9 KO SARM1 ASO. (**D**) Western blot of APP-FL and CTFs in control and NAD^+^ treated WT and KO neurons. (**E**) Quantification of APP-FL band expression relative to WT control. Data was analyzed using one-way ANOVA with Tukey’s multiple comparisons test. (**F**) Quantification of APP-CTFs band intensity relative to WT control. Data was analyzed using Kruskal–Wallis test with Dunn’s multiple comparisons test. WT Veh vs. KO Veh *, *p* = 0.0132. *n* = 9 WT, 9 WT + NAD, 8 KO, 9 KO + NAD. Samples were collected from three independent experiments. All bar graphs represent mean ± SEM.

APP accumulation phenotypes in NMNAT2 KO neurons have been shown to depend on SARM1 [25,26]. Loss of SARM1 has been reported to decrease amyloidogenic protein aggregation, supporting a connection between SARM1-driven metabolic stress responses and protein homeostasis [50]. To determine whether SARM1 KD reduces APP-CTF accumulations in NMNAT2 KO neurons, we treated WT and KO neurons with Ctrl- or SARM1-ASO from DIV1–12 as previously described [25] (Figure 2A). We found that APP-CTF levels in SARM1-ASO-treated KO neurons were restored to WT levels while APP-FL levels remained unchanged (Figure 2B,C). Notably, 1 mM NAD^+^ supplementation during DIV5–12 only partially reduces APP-CTF levels in NMNAT2 KO neurons (Figure 2D–F).

### 3.2. SARM1 KD Does Not Restore NAD^+^ Levels in NMNAT2 KO Neurons to WT Levels

**Figure 3:**
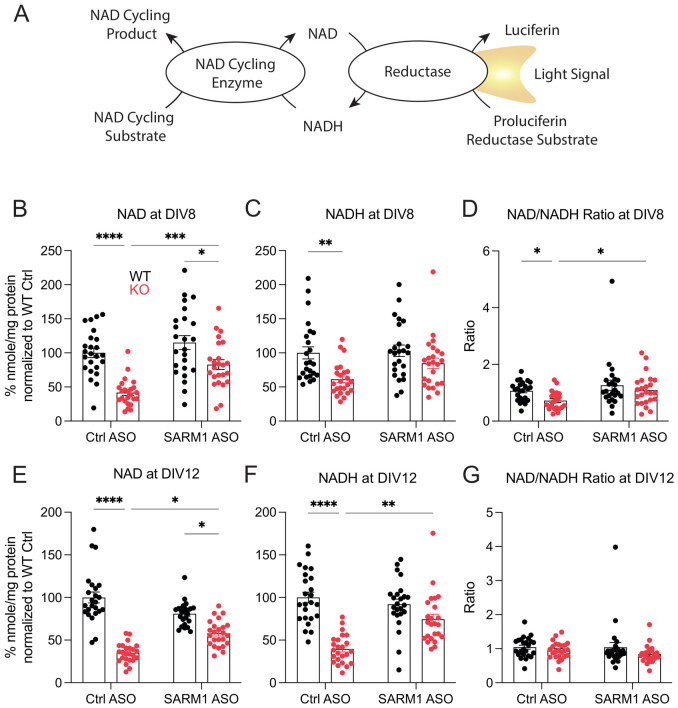
SARM1 knockdown partially restores NAD^+^ redox balance but not total NAD^+^ levels in NMNAT2 KO neurons. (**A**) Illustration of the reactions involved in the NAD^+^/NADH-Glo™ bioluminescent assay (**B**) NAD^+^ quantification following DIV1–8 of ASO treatment. Data was analyzed using two-way ANOVA with Tukey’s multiple comparisons test. *, *p* = 0.0136, ***, *p* = 0.0009, ****, *p* < 0.001. (**C**) NADH quantification following DIV1–8 of ASO treatment. Data was analyzed using Kruskal–Wallis test with Dunn’s multiple comparisons test. **, *p* = 0.002. (**D**) NAD^+^ /NADH ratio quantification following DIV1–8 of ASO treatment. Data was analyzed using Kruskal–Wallis test with Dunn’s multiple comparisons test. WT Ctrl ASO vs. KO Ctrl ASO *, *p* = 0.0139, KO Ctrl ASO vs. KO SARM1 ASO *, *p* = 0.0334. *n* = 24 WT Ctrl ASO, 24 WT SARM1 ASO, 24 KO Ctrl ASO, 24 KO SARM1 ASO. (**E**) NAD^+^ quantification following DIV1–12 of ASO treatment. Data was analyzed using Kruskal–Wallis test with Dunn’s multiple comparisons test. KO Ctrl ASO vs. KO SARM1 ASO *, *p* = 0.0182, WT SARM1 ASO vs. KO SARM1 ASO *, *p* = 0.0127, ****, *p* < 0.001. (**F**) NADH quantification following DIV1–12 of ASO treatment. Data was analyzed using Kruskal–Wallis test with Dunn’s multiple comparisons test. **, *p* = 0.0027, ****, *p* < 0.001. (**G**) NAD^+^ /NADH ratio quantification following DIV1–12 of ASO treatment. Data was analyzed using Kruskal–Wallis test with Dunn’s multiple comparisons test. *n* = 24 WT Ctrl ASO, 24 WT SARM1 ASO, 24 KO Ctrl ASO, 24 KO SARM1 ASO. Samples were collected from three independent experiments. All bar graphs represent mean ± SEM.

SARM1, a metabolic sensor activated by a reduced NAD^+^/NMN ratio following NMNAT2 loss, promotes further NAD^+^ depletion and axonal degeneration [27,28]. Our prior studies identified DIV8 as the earliest stage when axonal deficits emerge in NMNAT2 KO neurons [25]. We therefore quantified NAD^+^/NADH ratios at DIV8 and DIV12 NMNAT2 KO and WT neurons (Figure 3A). Consistent with prior observations, NMNAT2 KO neurons treated with control ASO exhibited significantly reduced NAD^+^, NADH, and NAD^+^/NADH ratios compared to WT at DIV8 (Figure 3B–D). SARM1 ASO treatment partially corrected this redox imbalance, restoring NADH levels and the NAD^+^/NADH ratio to near-control levels, but without fully rescuing NAD^+^ abundance (Figure 3B–D). At DIV12, despite significant increases in NAD^+^ and NADH levels in NMNAT2 KO neurons with SARM1 ASO than KO neurons with control ASO, NAD^+^ was not restored to control levels (Figure 3E–G). Together, these data indicate that SARM1 knockdown mitigates NAD^+^ depletion in NMNAT2-deficient neurons but is insufficient to fully restore NAD^+^ homeostasis.

### 3.3. Metabolic Functional Analyses Show the Importance of the NMNAT2–SARM1 Axis in Neuronal Glucose Metabolism

**Figure 4:**
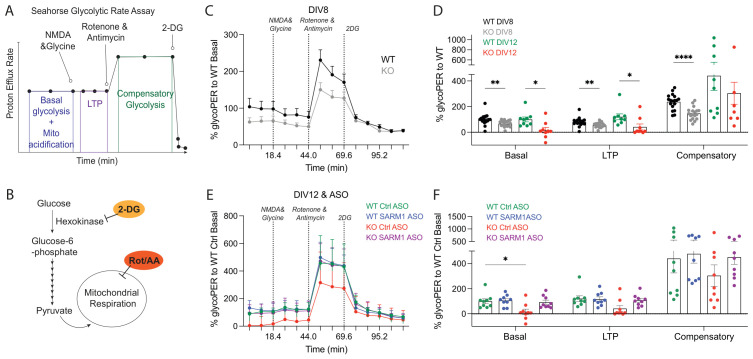
Downregulated glycolytic capacity in NMNAT2 KO neurons can be rescued by SARM1 KD. (**A**) Seahorse Induced Glycolytic Rate test profile. (**B**) Illustration of the glycolysis pathway and the targets for rotenone and antimycin, and 2DG. (**C**) Glycolytic rate assay response curve of NMNAT2 WT and KO neurons at DIV8. (**D**) Normalized glycoPER rates of DIV8 and DIV12 WT and KO neurons at basal, LTP, and compensatory steps. Data was analyzed using Mann–Whitney test and Holm–Šidák’s multiple comparisons test. DIV8 WT vs. KO Basal; **, *p* = 0.0026. DIV8 WT vs. KO LTP; **, *p* = 0.0021. DIV8 WT vs. KO Compensatory; ****, *p* < 0.001. DIV12 WT vs. KO Basal; *, *p* = 0.0106. DIV12 WT vs. KO LTP; *, *p* = 0.0142. *n* = 18 WT DIV8, 18 KO DIV8, 9 WT DIV12, 9 KO DIV12. DIV8 samples were collected from four independent batches and DIV12 samples were collected from two independent batches. (**E**) Glycolytic rate assay response curve of NMNAT2 WT and KO neurons treated with Ctrl or SARM1 ASO at DIV12. (**F**) Normalized glycoPER rates of DIV12 WT and KO neurons treated with ASO at basal, LTP, and compensatory steps. Data was analyzed using Kruskal–Wallis test with Dunn’s multiple comparisons test. *, *p* = 0.0459. *n* = 9 WT Ctrl ASO, 9 WT SARM1 ASO, 9 KO Ctrl ASO, 9 KO SARM1 ASO. Samples collected from two independent batches. All bar graphs represent mean ± SEM.

The NAD^+^/NADH redox potential is essential to drive glycolysis and oxidative phosphorylation. NAD^+^/NADH redox potentials dictate the rate of glycolytic flux, serve as vital electron acceptors, and are essential for maintaining glucose-derived ATP production [51]. To determine how NAD^+^ reduction in neurons impacts energy homeostasis, we first determined if glycolysis is impaired in NMNAT2 KO neurons and whether SARM1 KD or exogenous NAD^+^ supplementation rescues it. Glycolytic Rate Seahorse assay was conducted with DIV8 and DIV12 WT and KO neurons. Glycolytic Rate assay measures proton efflux rate (PER) at basal, induced, and compensatory stages, which is then transformed into glycoPER to assess cellular glycolytic capacity. First, we injected NMDA and glycine to induce chemical long-term potentiation (LTP) [52] and measured glycolysis under enhanced synaptic transmission (Figure 4A). Next, we measured compensatory responses by injecting a combination of rotenone and antimycin to block mitochondrial respiration and push the cells to their maximum glycolytic capacity. Finally, 2DG was injected to block glycolysis to confirm that the previous measurements were primarily derived from glycolysis [53] (Figure 4B).

We observed significantly reduced glycoPER responses in NMNAT2 KO neurons compared to WT through basal, LTP, and compensatory phases at DIV8 (Figure 4C,D). Similar reductions were observed with DIV12 KO neurons, except for compensatory responses. Next, we treated neurons with SARM1 ASO to determine if reducing SARM1 could restore glycolytic capacity. We found that treatment with SARM1 ASO KO restored glycoPER responses in all three phases to control levels, whereas Ctrl ASO treatment exerted no rescue (Figure 4E,F).

**Figure 5:**
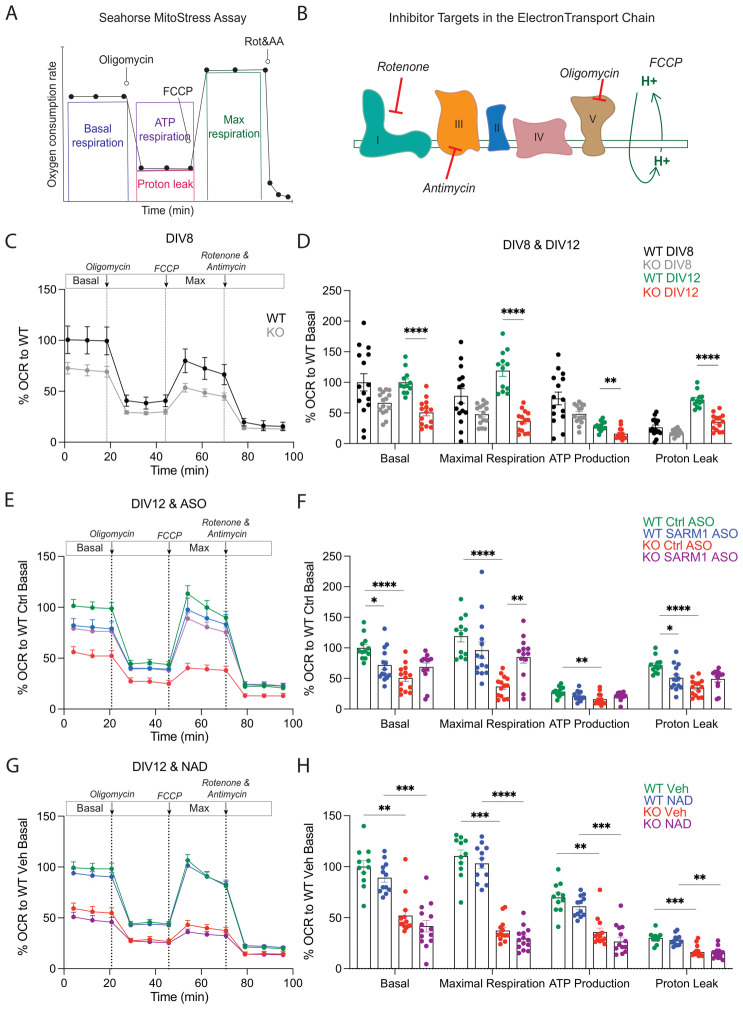
Decreased mitochondrial capacity in NMNAT2 KO neurons can be rescued by SARM1 KD but not by NAD^+^ supplementation. (**A**) Seahorse MitoStress Assay test profile. (**B**) Mitochondria respiratory chain complex and illustration of targets for rotenone, antimycin, oligomycin, and FCCP. (**C**) MitoStress assay response curve of NMNAT2 WT and KO neurons at DIV 8. (**D**) Normalized OCR rates of DIV8 and DIV12 WT and KO neurons at basal, maximal respiration, ATP production, and proton leak steps. Data was analyzed using Mann–Whitney test and Holm–Šidák’s multiple comparisons test. **, *p* = 0.0013, ****, *p* < 0.001. *n* = 15 WT DIV8, 15 KO DIV8, 12 WT DIV12, 14 KO DIV12. (**E**) MitoStress assay response curve of NMNAT2 WT and KO neurons treated with Ctrl or SARM1 ASO at DIV12. (**F**) Normalized OCR rates of DIV12 WT and KO neurons treated with ASO at basal, maximal, ATP production, and proton leak steps. Data was analyzed using Kruskal–Wallis test with Dunn’s multiple comparisons test. Basal; *, *p* = 0.0238, ****, *p* < 0.001. Maximal respiration; **, *p* = 0.0045, ****, *p* < 0.001. ATP production; **, *p* = 0.0022. Proton leak; *, *p* = 0.032, ****, *p* < 0.0001. *n* = 12 WT Ctrl ASO, 14 WT SARM1 ASO, 14 KO Ctrl ASO, 14 KO SARM1 ASO. (**G**) MitoStress assay response curve of NMNAT2 WT and KO neurons treated with vehicle or NAD^+^ at DIV12. (**H**) Normalized OCR rates of DIV12 WT and KO neurons treated with vehicle or NAD^+^ at basal, maximal respiration, ATP production, and proton leak steps. Data was analyzed using Kruskal–Wallis test with Dunn’s multiple comparisons test. Basal; **, *p* = 0.0013, ***, *p* = 0.0004. Maximal respiration; ***, *p* = 0.0004, ****, *p* < 0.001. ATP production; **, *p* = 0.0017, ***, *p* = 0.0003. Proton leak; **, *p* = 0.0012, ***, *p* = 0.0008. *n* = 11 WT Veh, 12 WT + NAD, 13 KO Veh, 14 KO + NAD. Samples collected from three independent batches. All bar graphs represent mean ± SEM.

SARM1 has been shown to localize in mitochondria [54,55]. To determine if mitochondrial function is impaired in NMNAT2 KO neurons, and whether SARM1 KD or NAD^+^ supplementation can restore its capacity, the MitoStress Seahorse assay was used to measure OCR at basal and maximal respiration conditions, and ATP production capacity (Figure 5A). The injection paradigm begins with oligomycin, which inhibits complex V of the electron transport chain, and the resulting drop in OCR provides information on the amount of ATP being produced by the mitochondria. This is followed by an injection of FCCP, a protonophore that uncouples the mitochondria’s proton gradient and stimulates rapid oxygen consumption to calculate maximal respiratory capacity in the mitochondria. Finally, a combination of rotenone and antimycin is injected to block complexes I and III of the electron transport chain and completely shut down the mitochondria, revealing OCR readings from proton leak [56] (Figure 5B).

Our data show that NMNAT2 KO neurons exhibit normal mitochondrial function at DIV8 (Figure 5C). However, at DIV12, OCR was significantly reduced in NMNAT2 KO neurons compared to WT controls across all test measurements (Figure 5D). SARM1 ASO treatment normalized the mitochondrial function of DIV12 KO neurons (Figure 5E,F), with similar OCR readings between SARM1-ASO-treated-NMNAT2 KO and -WT neurons for basal, maximum respiration, and ATP production phases. Ctrl ASO treatment did not rescue mitochondrial deficits in DIV12 NMNAT2 KO neurons. To our surprise, exogenous NAD^+^ supplementation did not rescue the mitochondrial function of DIV12 KO neurons (Figure 5G,H). Taken together, our data show that mitochondrial function is significantly impaired in NMNAT2 KO neurons at DIV12 in a SARM1-dependent manner.

### 3.4. Temporal Profiling of APP-CTF Accumulations and the Proteomic Profiles Revealed an Initial Progression Phase Followed by an Accelerating Phase

**Figure 6:**
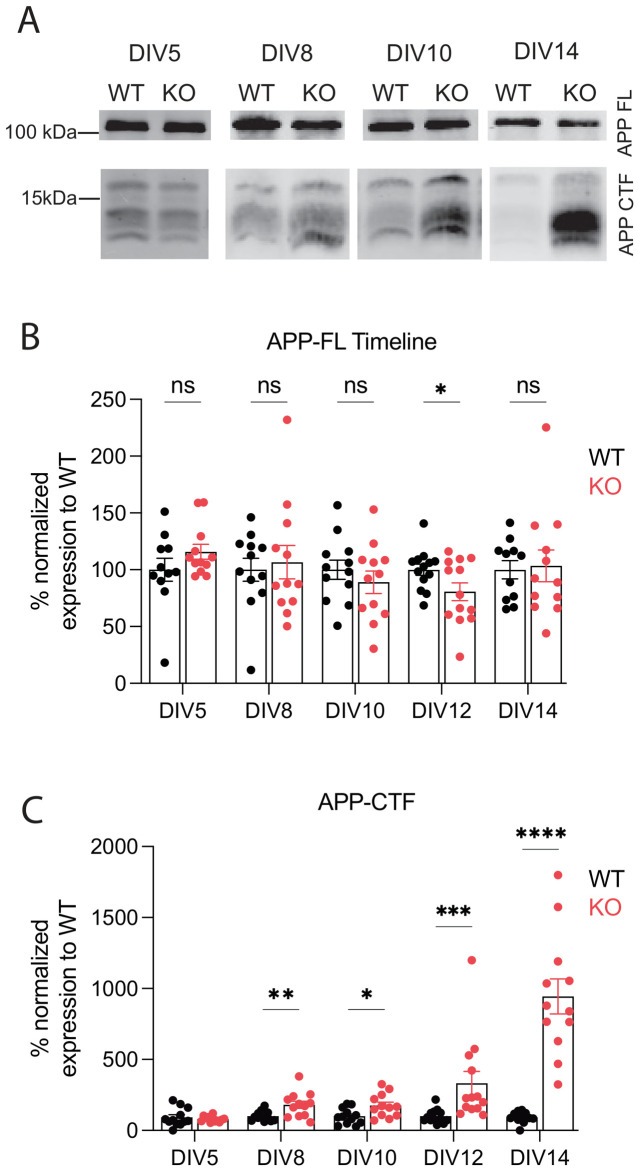
APP accumulation in NMNAT2 KO neurons has a biphasic expression pattern. (**A**) Western blot of WT and KO neuronal culture lysates at DIV5, 8, 10, and 14 showing APP-FL and CTF expression. (**B**) Western blot analysis of APP-FL expression relative to corresponding DIV WT neurons at DIV5, 8, 10, 12, and 14. DIV5; *n* = 11 WT, 12 KO; APP-FL was analyzed with unpaired t-test. DIV8; *n* = 12 WT, 12 KO; APP-FL was analyzed with Mann–Whitney test. DIV10; *n* = 12 WT, 12 KO; APP-FL was analyzed with unpaired *t*-test. DIV12; *n* = 13 WT, 13 KO; APP-FL was analyzed with unpaired *t*-test. *, *p* = 0.049, ns, not significant. DIV14; *n* = 11 WT, 12 KO; APP-FL was analyzed with Mann–Whitney test. Samples were collected from three independent experiments. (**C**) Western blot analysis of APP-CTF expression relative to corresponding DIV WT neurons at DIV5, 8, 10, 12, and 14. DIV5; *n* = 11 WT, 12 KO; APP-CTF was analyzed with Mann–Whitney test. DIV8; *n* = 12 WT, 12 KO; APP-CTF was analyzed with Mann–Whitney test. **, *p* = 0.0083. DIV10; *n* = 12 WT, 12 KO; APP-CTF was analyzed with unpaired *t*-test. *, *p* = 0.015. DIV12; *n* = 13 WT, 12 KO; APP-CTF was Mann–Whitney test. ***, *p* = 0.0002. DIV14; *n* = 11 WT, 12 KO; APP-CTF was analyzed with Mann–Whitney test. ****, *p* ≤ 0.0001. Samples were collected from three independent experiments. All bar graphs represent mean ± SEM.

To determine when APP-CTFs start to accumulate and the temporal progression in relationship to glycolysis and mitochondria dysfunction onsets, we examined the abundance of APP-FL and APP-CTFs in DIV5/8/10/14 WT and NMNAT2 KO neurons with Western blotting (Figure 6A). APP-FL did not differ between WT and KO neurons at these timepoints (Figure 6B). At DIV5, KO neurons showed normal levels of APP-CTFs ranging from 10–18 kDa (Figure 6C). By DIV8, APP-CTFs are significantly higher in KO neurons, and this difference accelerates between DIV12 and 14 (Figure 6C), when mitochondrial dysfunction is evident in KO neurons.

**Figure 7:**
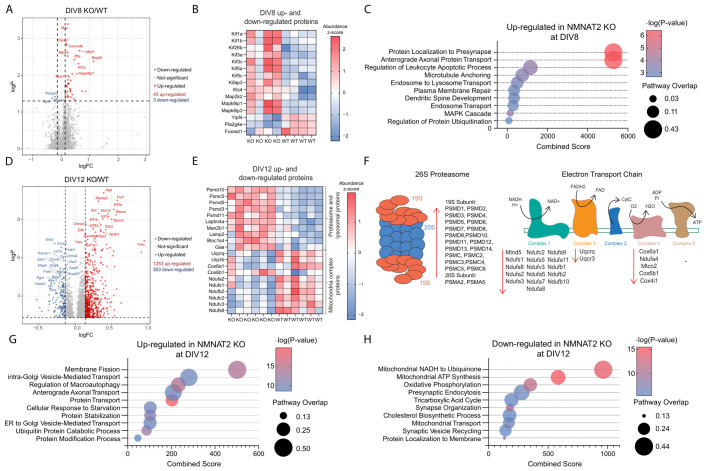
Proteomic analysis of NMNAT2 KO neurons reveals transport, proteostasis, and mitochondrial complex changes. (**A**) Volcano plot of abundance ratio for all identified genes at DIV8 in WT and KO samples from proteomic analysis. Significantly downregulated genes in KO samples are shown in blue (n = 3), and significantly upregulated genes are shown in red (n = 42). (**B**) Heatmap of protein abundance across DIV8 WT and KO samples. Values were z-scored per gene, with colors indicating relative abundance compared to each protein’s mean across samples (red > 0; blue < 0). (**C**) Bubble plot of enriched GO Biological processes from upregulated gene list at DIV8. (**D**) Volcano plot of abundance ratio for all identified genes at DIV12 in WT and KO samples from proteomic analysis. Significantly downregulated genes in KO neurons are shown in blue (n = 553), and significantly upregulated proteins are shown in red (n = 1253). (**E**) Heatmap of protein abundance in DIV12 WT and KO samples. Values were z-scored per gene, with colors indicating relative abundance compared to each protein’s mean across samples (red > 0; blue < 0). (**F**) Illustration of the 26S proteasome and its corresponding upregulated genes in NMNAT2 KO neurons at DIV12 (left) and illustration of mitochondria complexes along with their corresponding downregulated genes in NMNAT2 KO neurons at DIV12 (right). Arrow direction indicates down or upregulation of genes. (**G**) Bubble plot of enriched GO Biological processes from upregulated gene list at DIV12. (**H**) Bubble plot of enriched GO Biological processes from downregulated gene list at DIV12. DIV8 n = 4 WT, 4 KO. DIV12 n = 6 WT, 6 KO.

To explore the mechanisms affected by neuronal NAD^+^ reduction due to NMNAT2 loss, proteomic analyses were conducted on WT and NMNAT2 KO neurons at both DIV8, the stage when APP processing deficits begin to appear, and at DIV12, when APP-CTF accumulations enter the accelerated phase. Comparing KO neurons to WT controls at DIV8, we found only 42 significantly upregulated and three significantly downregulated proteins (Figure 7A). Among the upregulated proteins, multiple kinesin superfamily members (Kif1a, Kif1b, Kif5a, Kif5c, Kif3a, and Kif3c) were elevated, along with mitogen-activated protein kinase (MAPK) pathway components Mapk8ip1, Mapk8ip3, and Map2k2. Mapk8ip1 and Mapk8ip3 function as c-Jun N-terminal kinase (JNK) scaffold proteins that associate with kinesin motors, linking MAPK signaling to axonal transport machinery [57,58] (Figure 7B). GO enrichment analysis identified significant overrepresentation of biological processes related to protein localization, endosomal and lysosomal pathways, MAPK cascade signaling, microtubule organization, and dendritic development (Figure 7C). These findings indicate coordinated upregulation of proteins involved in intracellular transport, vesicular trafficking, cytoskeletal organization, and MAPK signaling at the onset of the phenotype.

At DIV12, the NMNAT2 KO neuronal proteomic profile was markedly different from that of WT controls, with 1253 upregulated and 533 downregulated proteins (Figure 7D). Notably, several coordinated changes were observed within distinct protein families and pathway-associated groups. Specifically, several proteasomal subunits were upregulated, whereas many components of the mitochondrial electron transport chain complexes were downregulated in NMNAT2 KO neurons (Figure 7E). Consistent with our metabolic assays, we found that seven of the 10 enzymes involved in the glycolysis pathway (Supplementary Figure S2A) were negatively correlated with APP abundance in our DIV12 samples (Supplementary Figure S2B). Additionally, correlation analyses of subunit proteins involved in oxidative phosphorylation revealed that the abundance of mitochondrial complexes I, II, and IV were negatively correlated with APP abundance upon NMNAT2 loss (Supplementary Figure S3A–E).

The 26S proteasome has 33 unique protein subunits, and 22 of these proteins were upregulated in NMNAT2 KO neurons (Figure 7F). Sixteen of the 45 mitochondria complex 1 subunit proteins, along with proteins associated with complex 3 and complex 4, were downregulated in NMNAT2 KO neurons (Figure 7F). GO enrichment analysis of upregulated proteins identified processes related to protein transport, macroautophagy, cellular response to starvation, and protein ubiquitination (Figure 7G); whereas downregulated proteins were enriched for pathways associated with mitochondrial function, oxidative phosphorylation, synaptic organization, and protein localization (Figure 7H).

Protein–protein interactions analyzed with STRING further supported the above pathways (Supplementary Figure S4). At DIV8, upregulated proteins clustered into a network associated with microtubule dynamics, kinesin complexes, and protein transport (Supplementary Figure S4A). At DIV12, upregulated proteins formed three major clusters linked to protein translation, protein folding, and ubiquitin-mediated proteasomal degradation (Supplementary Figure S4B), while downregulated proteins clustered within glycolysis, pyruvate metabolism, oxidative phosphorylation, and GTPase signaling networks (Supplementary Figure S4C). Collectively, these data suggest NMNAT2 loss results in alterations in transport machinery at DIV8, followed by compromised proteostatic and mitochondrial metabolic function by DIV12.

## 4. Discussion

The NMNAT2–SARM1 axis is a key regulator of neuronal NAD^+^ homeostasis and energy metabolism [25]. Here, we show that neuronal NMNAT2 loss induces SARM1-dependent accumulation of APP-CTF. Temporal profiling shows that APP-CTF accumulation in NMNAT2 KO neurons coincides with early reductions in NAD^+^/NADH redox potential and impaired glycolysis, while mitochondrial function is initially preserved but declines at later stages. This delayed mitochondrial dysfunction is accompanied by extensive proteomic remodeling and accelerated APP-CTF accumulation. Notably, SARM1 KD restores APP processing, glycolysis, and oxidative phosphorylation in NMNAT2 KO neurons, whereas NAD^+^ supplementation provides only partial rescue. Together, these findings support a model in which disruption of NAD^+^-dependent redox homeostasis drives defects in APP processing, positioning the NMNAT2–SARM1 axis as a central regulator linking neuronal bioenergetics to proteostasis.

### 4.1. Progressive Metabolic Failure Disrupts APP Processing

APP-CTFs begin to accumulate in NMNAT2 KO neurons at DIV8 (Figure 6), coinciding with previously reported axonal transport deficits in APP-containing cargos [25]. This timepoint corresponds to a stage of active synaptogenesis and increasing energetic demand in neurons [59,60]. The temporal alignment between rising energy demand, reduced NAD^+^ levels (Figure 3), and APP-CTF accumulation suggests that impaired energy homeostasis is a primary cause of defective APP processing.

Metabolic assays revealed significant glycolytic deficits at DIV8 (Figure 4), consistent with prior observations in NMNAT2-deficient axons [25]. In contrast, mitochondrial oxidative phosphorylation deficits emerge later at DIV12 (Figure 5), coinciding with a sharp increase in APP-CTF levels (Figure 6C). Proteomic analysis identified downregulation of mitochondrial complex I proteins in NMNAT2 KO neurons at DIV12 (Figure 7F), but not at DIV8, raising the possibility that progressive mitochondrial dysfunction amplifies defects in APP processing.

Mitochondrial dysfunction is linked to impaired mitochondrial protein synthesis and disrupted organelle maintenance [61]. This is consistent with the coordinated reduction in respiratory complex components observed in our proteomic data. In addition, mitochondrial dysfunction and abnormal APP processing can influence each other [62], potentially establishing a pathogenic feedback loop that exacerbates proteostatic stress and contributes to the accelerated accumulation of APP-CTFs after DIV12.

It is worth highlighting the potential role of Poly(ADP-ribose) polymerases (PARPs) in mitochondrial dysfunction. Oxidative stress and DNA damage are known activators of PARPs [63], a family of enzymes that catalyze the transfer of ADP-ribose from NAD^+^ onto protein substrates [64]. The overactivation of PARP-1 has been implicated in neurodegeneration, as its consumption of NAD^+^ leads to glycolytic inhibition and mitochondrial failure [65]. Oxidative stress in NMNAT2 KO neurons could activate PARPs, making it crucial to elucidate how this enzyme influences the metabolic phenotypes we observe.

The NAD^+^-dependent enzymes sirtuins, particularly SIRT1, combat DNA damage and mitochondria dysfunction by increasing mitochondria biogenesis through activation of peroxisome proliferator-activated receptor γ coactivator 1-α (PGC1α) [66,67,68]. NAD^+^ deficiency in NMNAT2 KO neurons could decrease sirtuin activity and subsequently reduce PGC1α [69], which can then lead to reduced transcriptional activity of peroxisome proliferator-activated receptors (PPARs) [70]. PPARγ promotes oxidative phosphorylation and mitochondrial biogenesis [71]. Future studies should be conducted to determine whether the reduced mitochondrial complex components observed in our DIV12 NMNAT2 KO neurons (Figure 7, Supplementary Figure S3) could be linked to reduced PPARγ activity.

### 4.2. APP Trafficking Defects Underlie Abnormal Processing in NMNAT2 KO Neurons

Proteomic profiling at DIV8 revealed upregulation of kinesin motor proteins and MAPK pathway components, including JNK scaffold proteins. JNK signaling, which is responsive to metabolic stress and regulates kinesin activity [72,73], may contribute to altered transport dynamics. Early APP axonal transport deficits observed in NMNAT2 KO axons [25] suggest that increased levels of axonal transport components may represent a compensatory response. However, altered motor protein expression and stress signaling could also impair trafficking through secretory and endocytic pathways, promoting APP retention in secretase-rich compartments and enhancing its cleavage [74,75]. Disrupted endosomal and trans-Golgi-network trafficking may further alter APP processing [76,77].

At later stages (DIV12), proteomic analysis revealed upregulation of unfolded protein response (UPR)-related proteins, including increased proteasome components and ubiquitin-mediated protein catabolism [78,79], consistent with ER stress [80]. These findings support a model in which NMNAT2 loss disrupts ER-to-Golgi trafficking and proteostatic balance. Additionally, the increase of APP-CTFs themselves may contribute to cellular stress, as CTF-99 fragments can impair lysosomal function, disrupt APP transport [36,81], and promote neuronal hyperexcitability [34]. Together, these results suggest that NMNAT2 loss leads to defects in protein trafficking and proteostasis, contributing to abnormal APP processing.

### 4.3. SARM1 Amplifies Metabolic and Proteostatic Dysfunction After NMNAT2 Loss

SARM1 activation caused by NMNAT2 loss amplifies metabolic failure via NAD^+^ consumption, contributing to downstream mitochondrial dysfunction [82,83]. SARM1 KD restores APP-CTF levels (Figure 2A–C), glycolytic function (Figure 4E,F), and mitochondrial oxidative capacity (Figure 5E,F), reversing both bioenergetic and proteostatic defects in NMNAT2 KO neurons despite incomplete normalization of NAD^+^ levels. This partial recovery is consistent with the continued absence of NMNAT2, the major NAD^+^ synthesizing enzyme in neurons. SARM1 KD prevents further pathological NAD^+^ consumption but does not replenish NAD^+^ pools. Our previous work shows NAD^+^ supplementation rescues axonal glycolysis [25]. Here, we found NAD^+^ partially rescued APP-CTF levels but failed to restore mitochondrial respiration, potentially reflecting compartmental limitations or incomplete correction of intracellular redox balance. These findings suggest that SARM1-mediated pathology extends beyond NAD^+^ depletion alone and involves a broader disruption of metabolic and redox homeostasis. The effective rescue by SARM1 KD demonstrates that blocking this amplification step enables neurons to endure suboptimal energy conditions.

## 5. Conclusions

In summary, loss of NMNAT2 triggers a progressive, SARM1-dependent disruption of neuronal redox homeostasis, energy metabolism, and proteostasis, leading to accumulation of APP-CTFs. This study advances our understanding of the molecular mechanisms underlying neurodegeneration by establishing a functional link among NAD^+^ metabolism, the NMNAT2–SARM1 pathway, and APP processing. By identifying NMNAT2 deficiency as a driver of aberrant APP metabolism, this work provides mechanistic insight into how disruptions in axonal maintenance contribute to neurodegenerative pathology. APP-CTFs are increasingly recognized as pathogenic mediators in both Alzheimer’s disease and related disorders, and our findings suggest that progressive impairment of proteostatic pathways may represent an early event linking axonal dysfunction to abnormal APP accumulation and neuronal vulnerability.

Our findings extend the role of the NMNAT2–SARM1 axis beyond axon degeneration, identifying it as a key regulator of neuronal proteostasis. By linking SARM1 activation to both mitochondrial dysfunction and aberrant APP processing, these results support a model in which metabolic and proteostatic stress act together to promote neuronal dysfunction. Notably, neurons remain resilient to glycolytic deficits when Sarm1 is suppressed, indicating that the initial metabolic stress is insufficient to trigger neurodegenerative pathways at reduced SARM1 levels. Together, these findings suggest that SARM1 inhibition may provide broad neuroprotection by preserving NAD^+^-dependent metabolic function while limiting proteostatic stress. Because disruption in NAD^+^ metabolism and proteostasis are hallmarks of numerous neurodegenerative diseases, the mechanisms described here may have implications well beyond APP-related pathology.

Future studies should determine whether enhancing mitochondrial function or redox balance can prevent proteinopathy and whether SARM1 is required for activation of stress pathways such as the UPR and upregulation of the ubiquitin–proteasome system.

## Data Availability

The original data presented in the study are openly available in MassIVE at https://doi.org/10.25345/C5610W61N or MSV000101981.

## Supplementary Materials

The following supporting information can be downloaded at: https://www.mdpi.com/article/10.3390/cells15121100/s1, Supplementary Figure S1: APP processing phenotype is not driven by BACE1 secretase activity. (A) Treatment timeline and western blot of DIV10-13 control (WT and KO) and beta-secretase inhibitor-treated neurons (WT + B-inh and KO + B-inh) showing APP-FL and CTF expression. (B) Quantification of western blot expression for APP-FL, (C) CTF cluster of bands, (D) CTF99 band, and (E) CTF83 band. n = 9 WT, 9 WT + B-inh, 9 KO, 9 KO + B-inh. APP-FL and CTF data was analyzed using one-way ANOVA with Tukey’s multiple comparisons test. APP-FL WT Veh vs. WT B-inh **, *p* = 0.0058. APP-FL KO Veh vs. KO B-inh **, *p* = 0.0048. APP-CTF WT Veh vs. KO Veh **, *p* = 0.0024. APP-CTF WT B-inh vs. KO B-Inh ***, *p* = 0.0001. APP-CTF99 and CTF83 data was analyzed using Mann-Whitney test. APP-CTF99 WT Veh vs. KO Veh **, *p* = 0.004. APP-CTF83 WT Veh vs. KO Veh **, *p* = 0.0012. APP-CTF83 WT B-inh vs. KO B-inh ****, *p* ≤ 0.001. Samples were collected from 3 independent experiments. All bar graphs represent mean ± SEM; Supplementary Figure S2: Correlation between the protein abundance of individual glycolytic enzymes and APP detected in the same samples by LC-MS. (A) Diagram illustrating the glucose metabolic pathway, with the enzymes for each step shown in red. (B) Summary plots show the correlations between individual enzymes and APP z-scores in WT and KO samples. Samples: 6 WT and 6 KO proteomic data. The correlations were analyzed using Simple Linear Regression; Supplementary Figure S3: Correlation between the protein abundance of OXPHOS complex proteins and APP detected in the same samples by LC-MS. (A) Combined abundance z-score for complex I components, including Ndufv3, Ndufc2, Ndufb2, Ndufa3, Ndufa2, Ndufb1, Ndufa11, Ndufa8, Ndufs1, Ndufa9, Ndufs5, Ndufb10, Ndufs8, Ndufa7, Ndufa5, Ndufs4, Ndufb11, Ndufs6, Ndufs2, Ndufb7, Ndufa13, Ndufb4, Ndufv1, Ndufb5, Ndufs3, Ndufb9, Ndufs7, Ndufv2, Ndufa12, Ndufa10, Ndufa1, Ndufa6, Ndufaf8, Ndufb3, Ndufaf4, Ndufv3, Ndufb6, Ndufb8 and Ndufaf3. (B) Combined abundance z-score for complex II components, including Sdhc, Sdhd, Sdhb and Sdha. (C) Combined abundance z-score for complex III proteins, including Uqcrc, Uqcrb, Uqcrh, Uqcrc1, Uqcrc2, Uqcrfs1 and Uqcr10. (D) Combined abundance z-score for complex IV components, including Ndufa4, Cox6b1, Cox6a1, Cox4i1, Cox7a2l, Cox7c, Cox5b, Cox5a, Cox6c, Cox7a2 and Mtco2. (E) Combined abundance z-score for complex V components, including Atp5e, Atp5h, Atp5j2, Atp5o, Atp5c1, Atp5c1, Atp5f1, Atp5a1, Atp5s, Atp5i, Atp5d, Atp5j, Atp5l, Atp5g1, and Atp5b. Samples: 6 WT and 6 KO proteomic data. The correlations were analyzed using Simple Linear Regression; Supplementary Figure S4: Interaction networks for up- and down-regulated proteins in NMNAT2 KO neurons at DIV8 and DIV12. (A) STRING cluster of up-regulated pathways in DIV8 KO neurons. (B) STRING clusters of up-regulated pathways in KO neurons at DIV12. (C) STRING clusters of down-regulated pathways in KO neurons at DIV12. Cytoscape’s STRING functional enrichment was performed to identify cluster pathways in all clusters, and FDR value was used to pick top terms for GO cellular component, molecular function, and/or biological process. DIV8 n = 4 WT, 4KO. DIV12 n = 6 WT, 6 KO; Supplementary Method: Mass spec Sample Preparation and LC-MS/MS/Data Analysis/Data Evaluation/Verification and Methods Write-Up.

## Abbreviations

The following abbreviations are used in this manuscript:

2DG	2-Deoxy-D-glucose
Aβ	Amyloid-Beta
AD	Alzheimer’s Disease
AICD	Amyloid Precursor Protein Intracellular Domain
APP	Amyloid Precursor Protein
APP-CTF	Amyloid Precursor Protein C-terminal Fragment
APP-FL	Full Length Amyloid Precursor Protein
ASO	Antisense Oligonucleotide
B-Inh	Beta-secretase Inhibitor
BACE1	Beta-site APP Cleaving Enzyme 1
BP	Biological Process
CC	Cellular Compartment
CTF-83	C-terminal Fragment 83
CTF-99	C-terminal fragment 99
DIV	Days In Vitro
ECAR	Extracellular Acidification Rate
FCCP	Carbonyl cyanide-4-(trifluoromethoxy)phenylhydrazone
glycoPER	Glycolytic Proton Efflux Rate
GO	Gene Ontology
HD	Huntington’s Disease
JNK	c-Jun N-terminal kinase
KD	Knockdown
kDa	Kilodalton
KO	Knockout
LC-MS	Liquid Chromatography-Mass Spectrometry
LTP	Long Term Potentiation
MAPK	Mitogen-activated Protein Kinase
MF	Molecular Function
NAD^+^	Nicotinamide Adenine Dinucleotide
NMDA	N-methyl-D-aspartic acid
NMNAT2	Nicotinamide Mononucleotide Adenylyltransferase 2
OCR	Oxygen Consumption Rate
PARPs	Poly(ADP-ribose) polymerases
PGC1α	Peroxisome Proliferator-Activated Receptor γ Coactivator 1-α
PPAR	Peroxisome Proliferator-Activated Receptor
PER	Proton Efflux Rate
PD	Parkinson’s Disease
Rot/AA	Rotenone/Antimycin A
sAPPα	Secreted APP-alpha
sAPPβ	Secreted APP-beta
SARM1	Sterile Alpha and TIR Motif Containing 1
SDS	Sodium Dodecyl Sulfate
UPR	Unfolded Protein Response
V	Volts
WT	Wildtype
