# Supplementary material for: Neurodegenerative NMNAT2 Deficiency Promotes APP Processing in a SARM1-Dependent Manner
Source: Cells. 2026 Jun 17;15(12):1100. doi: 10.3390/cells15121100 (PMC13297387; doi:10.3390/cells15121100)
Supplement: Supplementary file 1 [file cells-15-01100-s001.zip › LC-MS Supplementary Method.pdf]

2022_04_80 and 2022_07_134 Hui-Chen Lu

Mass spec Sample Preparation

Emma H Doud [1-2],

LC-MS/MS/ Data Analysis/Data Evaluation/Verification and Methods Write-Up

Emma H. Doud[1, 2]; Amber L. Mosley[1-2]

[1]Biochemistry, Molecular Biology and Pharmacology; [2]Center for Proteome Analysis; Indiana University School of Medicine (IUSM), 635 Barnhill Drive, Medical Science Building 0034, Indianapolis, IN 46202-5122, U.S.A.

Sample preparation, mass spectrometry analysis, bioinformatics, and data evaluation for quantitative proteomics and phosphoproteomics experiments were performed in collaboration with the Indiana University Proteomics Center for Proteome Analysis at the Indiana University School of Medicine similarly to previously published protocols. (Grecco GG, Huang JY, Muñoz B, Doud EH, Hines CD, Gao Y, Rodriguez B, Mosley AL, Lu HC, Atwood BK. Sex-Dependent Synaptic Remodeling of the Somatosensory Cortex in Mice With Prenatal Methadone Exposure. Advances in Drug and Alcohol Research. 2. PMID 37829495 DOI: 10.3389/adar.2022.10400)

Sample Preparation

Flash frozen cell pellets were lysed and extracted in 8 M urea, 50 mM Tris pH 8.5 using a Bioruptor® sonication system (Diagenode Inc. USA, North America cat number B01020001) with 30 sec/30 sec on/off cycles for 30 minutes in a water bath at 4 ºC. After subsequent centrifugation at 14,000 rcf for 20 min, protein concentrations were determined by Bradford protein assay (BioRad Cat No: 5000006). 35 µg (Project 1) or 50 ug (Project 2) equivalent of protein from each sample were then treated with 5 mM tris(2-carboxyethyl)phosphine hydrochloride (Sigma-Aldrich Cat No: C4706) to reduce disulfide bonds and the resulting free cysteine thiols were alkylated with 10 mM chloroacetamide (Sigma Aldrich Cat No: C0267). Samples were diluted with 50 mM Tris.HCl pH 8.5 (Sigma-Aldrich Cat No: 10812846001) to a final urea concentration of 2 M for overnight Trypsin/Lys-C digestion at 35 ºC (1:50 protease:substrate ratio, Mass Spectrometry grade, Promega Corporation, Cat No: V5072.)

Peptide Purification and Labeling

Digestions were acidified with trifluoroacetic acid (TFA, 0.5% v/v) and desalted on Waters Sep-Pak® Vac cartridges (Waters™ Cat No: WAT054955) with a wash of 1 mL 0.1% TFA followed by elution in 0.6 mL of 70% acetonitrile 0.1% formic acid (FA). Peptides were dried by speed vacuum and resuspended 50 mM triethylammonium bicarbonate. Peptide concentrations were checked by Pierce Quantitative colorimetric assay (Cat No: 23275). The same amount of peptide from each sample was then labeled for two hours at room temperature, with 0.25 mg of Tandem Mass Tag Pro (TMTpro) reagent (16-plex kit, manufactures instructions Thermo Fisher Scientific, TMTpro™ Isobaric Label Reagent Set; Cat No: 44520, lot no. VL313890 for WTvs KO and lot WG33375 for Div8 WT vs KO see Table X below) (Li et al., 2020). After checking to confirm >98% labeling efficiency, reactions were quenched with 0.3 % hydroxylamine (v/v) at room temperature for 15 minutes. Labeled peptides were then mixed and dried by speed vacuum.

2022-04-80 TMTpro labeling

ID	TMTpro label
WT3-25	127N
WT4-25	127C
WT5-25	128N
WT3-29	128C
WT4-29	129N
WT5-20	129C
KO3-25	130N

KO4-25	130C
KO5-25	131N
KO3-29	131C
KO4-29	132N
KO5-29	132C

2022_07_134 TMTpro labeling

Sample ID	TMTpro label
5-23 DIV8 WT#3 P3	126
5-23 DIV8 KO#3 P3	127N
6-6 DIV8 WT#1 P5	127C
6-6 DIV8 KO#1 P5	128N
5-23 DIV8 WT+NMN#1 P3	128C
5-23 DIV8 KO+NMN#1 P3	129N
6-6 DIV8 WT+NMN#1 P5	129C
6-6 DIV8 WT+NMN#2 P5	130N
6-6 DIV8 KO+NMN#1 P5	130C
6-6 DIV8 KO+NMN#2 P5	131N
8-6 DIV8 WT#1	131C
8-6 DIV8 KO#1	132N
8-6 DIV8 WT#2	132C
8-6 DIV8 KO#2	133N
8-6 DIV8 WT+NMN#1	133C
8-6 DIV8 KO+NMN#1	134
8-6 DIV8 WT+NMN#2	134C
8-6 DIV8 KP+NMN#2	135N

High pH Basic Fractionation and LC-MS/MS for 2022_04_80

After resuspending in 0.1% TFA, approximately one third of the TMTpro labeled peptide mix was fractionated on Sep-Pak® Vac cartridges using methodology and reagents from Pierce™ High pH reversed-phase peptide fractionation kit (Fractions with 12.5%, 15%, 17.5%, 22%, 22.5%, 25%, 35%, and 70% acetonitrile in 0.1% triethylamine, Thermo Fisher Cat No: 84868).

Nano-LC-MS/MS for 2022_04_80

Mass spectrometry was performed utilizing an EASY-nLC 1200 HPLC system (SCR: 014993, Thermo Fisher Scientific) coupled to Exploris 480™ mass spectrometer with FAIMSpro interface (Thermo Fisher Scientific). 1/5th of each fraction was loaded onto a 25 cm EasySpray column (Thermo Fisher Scientific) at 400 nL/min. The gradient (Mobile phases A: 0.1% formic acid (FA), water; B: 0.1% FA, 80% Acetonitrile (Thermo Fisher Scientific Cat No: LS122500)), was head at 6% B for 5 minutes, increased from 6-32%B over 160 minutes; 32-80% B over 10 mins; and dropping from 80-6% B over the final 5 min. The mass spectrometer was operated in positive

ion mode, default charge state of 2, advanced peak determination on, and lock mass of 445.12003. Three FAIMS CVs were utilized (-40 CV; -55 CV; -70CV) each with a cycle time of 1.3 s and with identical MS and MS2 parameters. Precursor scans (m/z 375-1500) were done with an orbitrap resolution of 60000, RF lens% 40, 50 ms maximum inject time, normalized AGC target of 300%, minimum MS2 intensity threshold of 5e4, MIPS mode to peptide, including charges of 2 to 8 for fragmentation with 60 sec dynamic exclusion. MS2 scans were performed with a quadrupole isolation window of 0.7 m/z, 32% HCD CE, 45000 orbitrap resolution, 200% AGC target, 120 ms maximum IT, fixed first mass of 100 m/z.

High pH Basic Fractionation and LC-MS/MS for 2022_07_134

The combined samples were resuspended in 10 mM ammonium formate, pH 10 half of each mix was fractionated using an offline Thermo UltiMate 3000 HPLC with a Waters Xbridge C18 column (3.5 µm x 4.6mm x 250 mm, Cat No: 186003943; Buffer A: 10 mM formate pH 10, Buffer B: 10 mM formate pH 10, 95% acetonitrile, gradient 1 mL/min 0-15%B over 5 min, 15-20% B over 5 min, 20-35%B over 75 min, 35-50% B over 5 min, 50-60% B over 10 min and a 6 minute hold at 60% B). Fractions were collected continuously every 60 seconds into 96 well plates. Initial and late fractions with minimal material were combined and lyophilized. The remaining fractions were concatenated into 24 fractions, dried down, and resuspended in 50 µL 0.1% FA prior to online LC-MS.

Nano-LC-MS for 2022_07_134

Samples were run (1/10th of each global fraction) on an EASY-nLC 1200 HPLC system (SCR: 014993, Thermo Fisher Scientific) coupled to an Eclipse Orbitrap™ mass spectrometer with FAIMSpro interface (Thermo Fisher Scientific). Each multiplex was run on a 25 cm Aurora Ultimate TS column (Ion Opticks Cat No: AUR2-25075C18) in a 45 °C column oven with a 180-minute gradient. The gradient was run from 5-30%B over 160 minutes; 30-90% B over 10 mins;; and dropping from 90-10% B over the final 10 min (Mobile phases A: 0.1% formic acid (FA), water; B: 0.1% FA, 80% Acetonitrile (Thermo Fisher Scientific Cat No: LS122500)). The mass spectrometer was operated in positive ion mode, default charge state of 2, advanced peak determination on, and Easy IC™ on. Four FAIMS CVs were utilized (-35V,-45 CV; -55 CV; -70CV) each with a cycle time of 0.6 s with identical MS and MS2 parameters. Precursor scans (m/z 400-1400) were done with an orbitrap resolution of 120000, RF lens% 30, 50 ms maximum inject time, standard automatic gain control (AGC) target, minimum MS2 intensity threshold of 4e4, MIPS mode to peptide, including charges of 2 to 7 for fragmentation with 60 sec dynamic exclusion shared across the cycles, excluding isotopes. MS2 scans were performed with a quadrupole isolation window of 0.7 m/z, 34% HCD collision energy, 50000 resolution, 250% AGC target, 120 ms maximum IT, fixed first mass of 100 m/z.

Mass spectrometry Data Analysis

Resulting RAW files were analyzed in Proteome Discover™ 2.5.0.400 (Thermo Fisher Scientific) with a *Mus musculus* UniProt reference proteome reviewed and unreviewed sequences FASTA plus common laboratory contaminants (downloaded 01092017, 49922 sequences). SEQUEST HT searches were conducted with both full trypsin digest and semi tryptic digest for identification of additional specific amyloid precursor protein peptides, 2 maximum number missed cleavages; precursor mass tolerance of 10 ppm; and a fragment mass tolerance of 0.02 Da. Static modifications used for the search were, 1) carbamidomethylation on cysteine (C) residues; 2) TMTpro label on N-termini of peptides and 3)

TMTpro label on lysine (K) residues. Dynamic modifications used for the search were oxidation of methionines, deamidation of asparagine, phosphorylation on serine, threonine or tyrosine, and acetylation, methionine loss or acetylation with methionine loss on protein N-termini. Percolator False Discovery Rate was set to a strict setting of 0.01 and a relaxed setting of 0.05. Values from both unique and razor peptides were used for quantification. In the consensus workflows, peptides were normalized by total peptide amount with no scaling. Quantification methods utilized TMTpro isotopic impurity levels available from Thermo Fisher Scientific. Reporter ion quantification was allowed with S/N threshold of 5 and co-isolation threshold of 50%. Resulting grouped abundance values for each sample type, abundance ratio values; and respective p-values (ANOVA (individual proteins)) for protein and peptide level abundances were exported from Proteome Discover to Microsoft Excel.
