# Supplementary material for: Neurodegenerative NMNAT2 Deficiency Promotes APP Processing in a SARM1-Dependent Manner
Source: Cells. 2026 Jun 17;15(12):1100. doi: 10.3390/cells15121100 (PMC13297387; doi:10.3390/cells15121100)
Supplement: Supplementary file 1 [file cells-15-01100-s001.zip › Supp Figure S4.pdf]

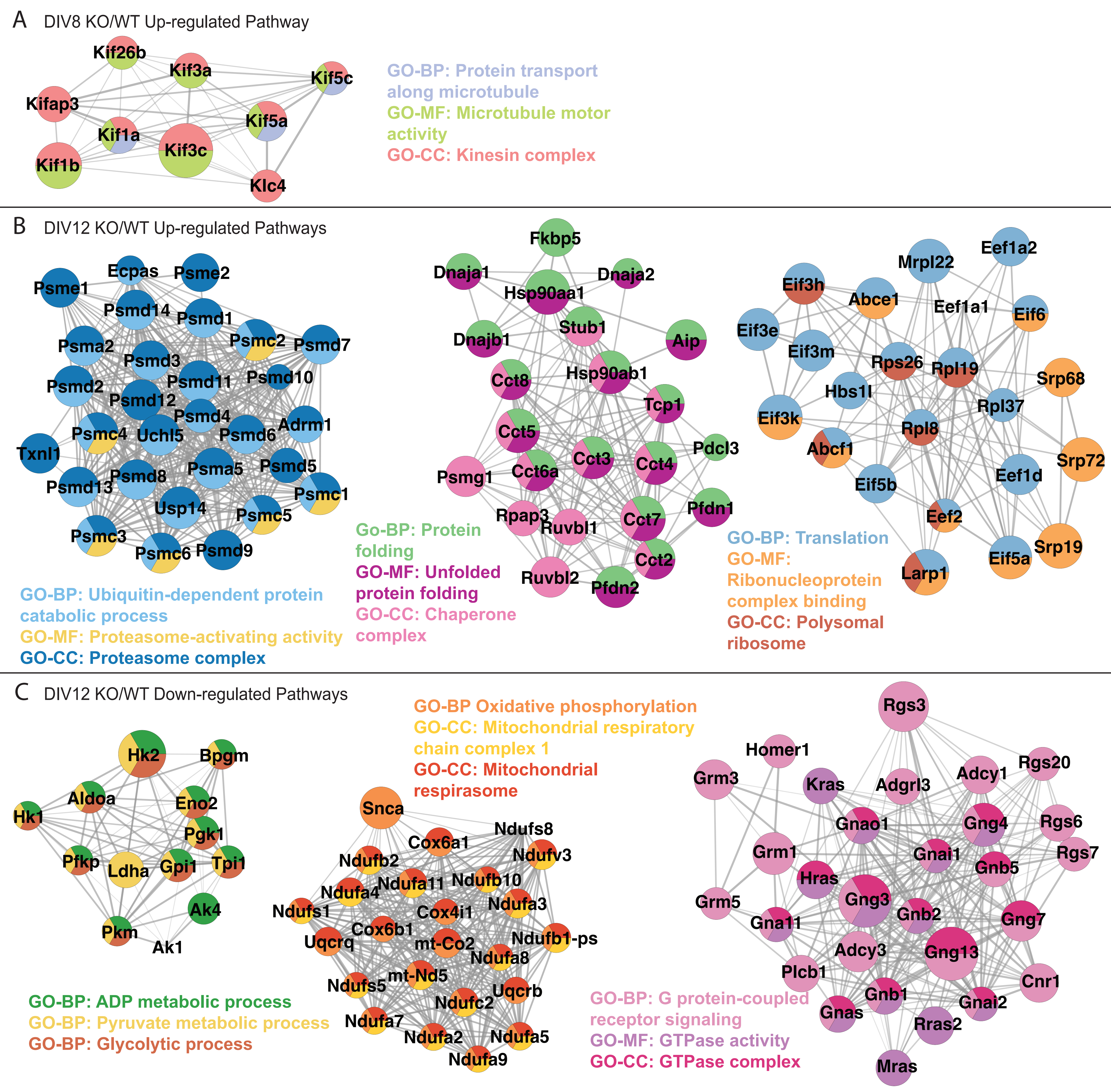
A
DIV8 KO/WT Up-regulated Pathway
Kif26b
Kif3a
Kif5c
Kifap3
Kif5a
Kif1a
Kif3c
Kif1b
Klc4
GO-BP: Protein transport along microtubule
GO-MF: Microtubule motor activity
GO-CC: Kinesin complex
B
DIV12 KO/WT Up-regulated Pathways
Ecpas
Psme2
Psme1
Psmd14
Psmd1
Psmc2
Psmd7
Psma2
Psmd3
Psmd11
Psmd10
Psmd2
Psmd12
Psmd4
Adrm1
Psmc4
Uchl5
Psmd6
Txnl1
Psma5
Psmd5
Psmd13
Psmd8
Psmc1
Usp14
Psmc5
Psmc3
Psmc6
Psmd9
GO-BP: Ubiquitin-dependent protein catabolic process
GO-MF: Proteasome-activating activity
GO-CC: Proteasome complex
Fkbp5
Dnaja1
Dnaja2
Hsp90aa1
Stub1
Dnajb1
Aip
Cct8
Hsp90ab1
Tcp1
Cct5
Pdcl3
Cct3
Cct4
Cct6a
Psmg1
Pfdn1
Rpap3
Ruvbl1
Cct7
Cct2
Ruvbl2
Pfdn2
Go-BP: Protein folding
GO-MF: Unfolded protein folding
GO-CC: Chaperone complex
Eef1a2
Mrpl22
Eif3h
Abce1
Eef1a1
Eif6
Eif3e
Eif3m
Rps26
Rpl19
Srp68
Hbs1l
Eif3k
Rpl37
Rpl8
Abcf1
Srp72
Eef1d
Eif5b
Eef2
Srp19
Eif5a
Larp1
GO-BP: Translation
GO-MF: Ribonucleoprotein complex binding
GO-CC: Polysomal ribosome
C
DIV12 KO/WT Down-regulated Pathways
Hk2
Bpgm
Aldoa
Eno2
Hk1
Pgk1
Pfkp
Ldha
Gpi1
Tpi1
Ak4
Pkm
Ak1
GO-BP: ADP metabolic process
GO-BP: Pyruvate metabolic process
GO-BP: Glycolytic process
GO-BP Oxidative phosphorylation
GO-CC: Mitochondrial respiratory chain complex 1
GO-CC: Mitochondrial respirasome
Snca
Ndufs8
Cox6a1
Ndufv3
Ndufb2
Ndufb10
Ndufa11
Ndufa4
Ndufa3
Ndufs1
Cox4i1
Cox6b1
Ndufb1-ps
Uqcrq
mt-Co2
Ndufa8
mt-Nd5
Ndufs5
Uqcrb
Ndufc2
Ndufa7
Ndufa2
Ndufa5
Ndufa9
Rgs3
Homer1
Rgs20
Grm3
Kras
Adgrl3
Adcy1
Rgs6
Gnao1
Gng4
Grm1
Gnai1
Rgs7
Hras
Gnb5
Gng3
Grm5
Gna11
Gnb2
Gng7
Adcy3
Gng13
Plcb1
Cnr1
Gnb1
Gnai2
Gnas
Rras2
Mras
GO-BP: G protein-coupled receptor signaling
GO-MF: GTPase activity
GO-CC: GTPase complex
