# Supplementary figures and images for: Neurodegenerative NMNAT2 Deficiency Promotes APP Processing in a SARM1-Dependent Manner
Source: Cells. 2026 Jun 17;15(12):1100. doi: 10.3390/cells15121100 (PMC13297387; doi:10.3390/cells15121100)

**A**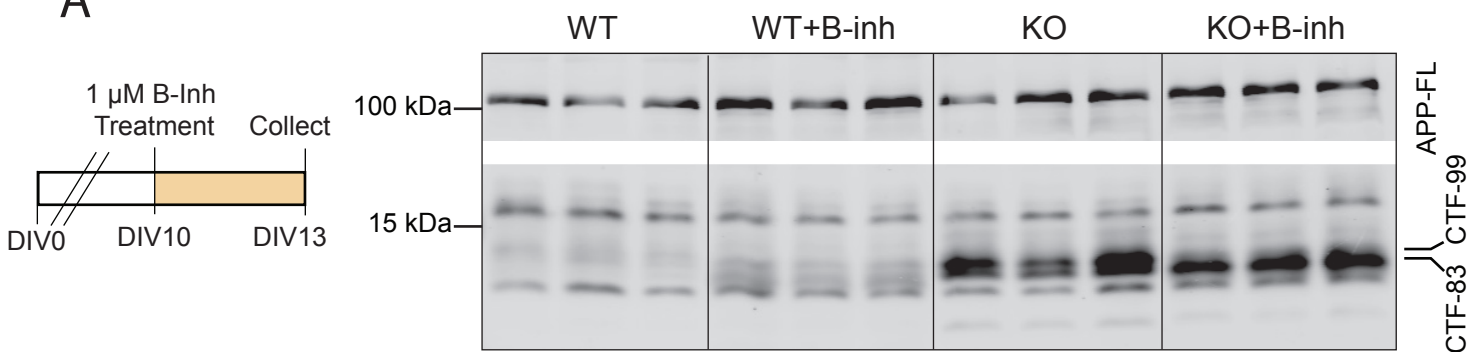**B**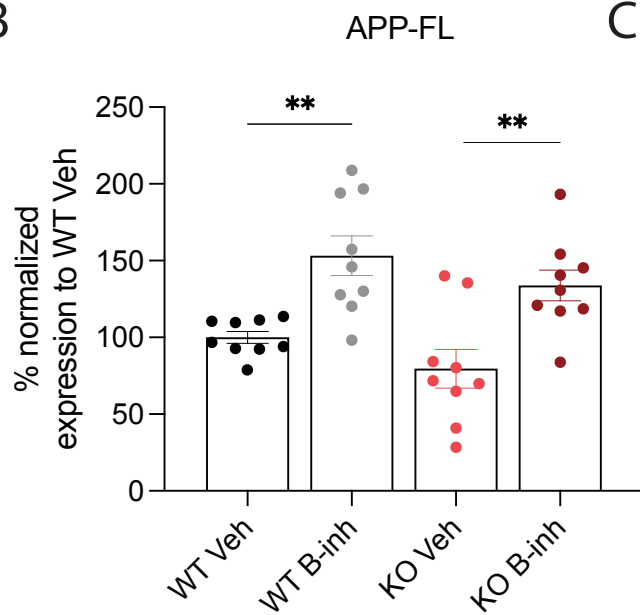**C**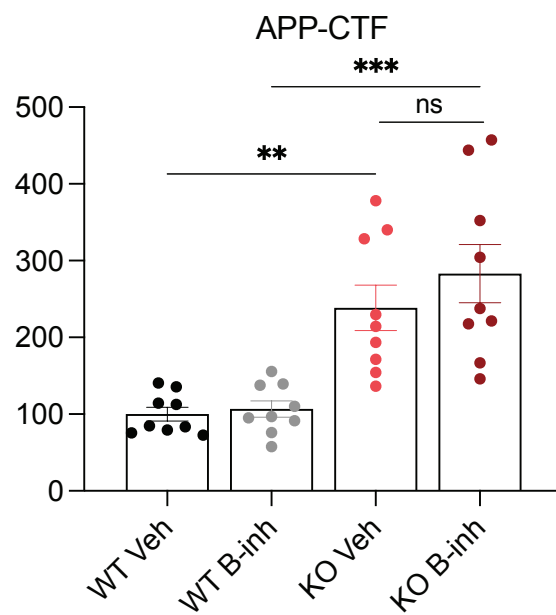**D**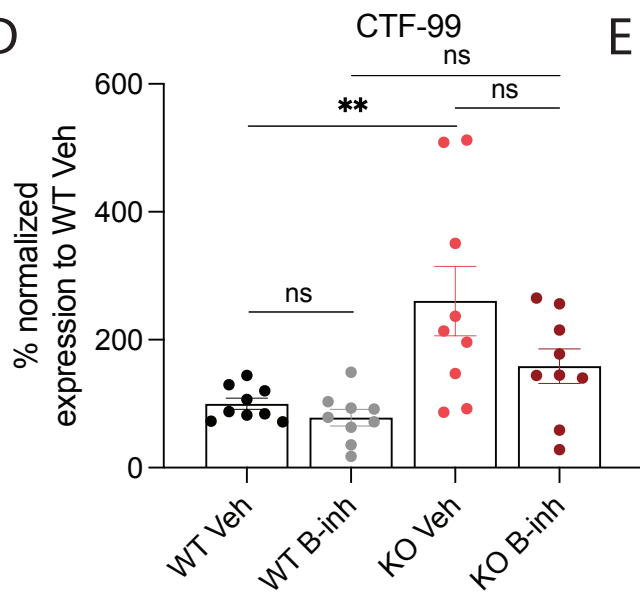**E**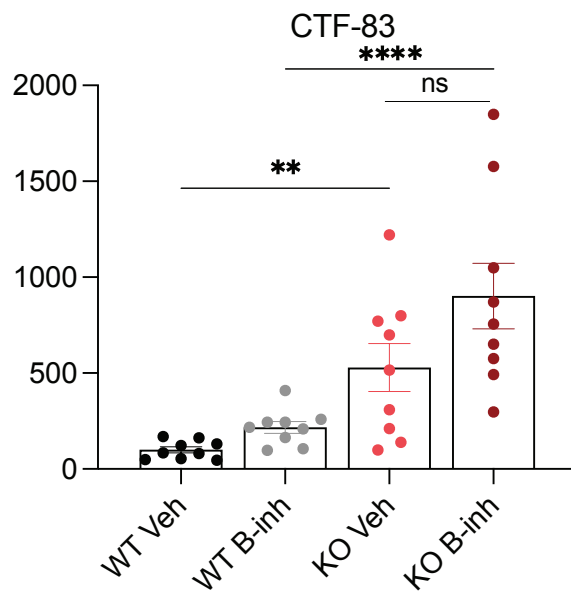

Supplement: Supplementary file 1 [file cells-15-01100-s001.zip › Supp Figure S1.pdf]

A

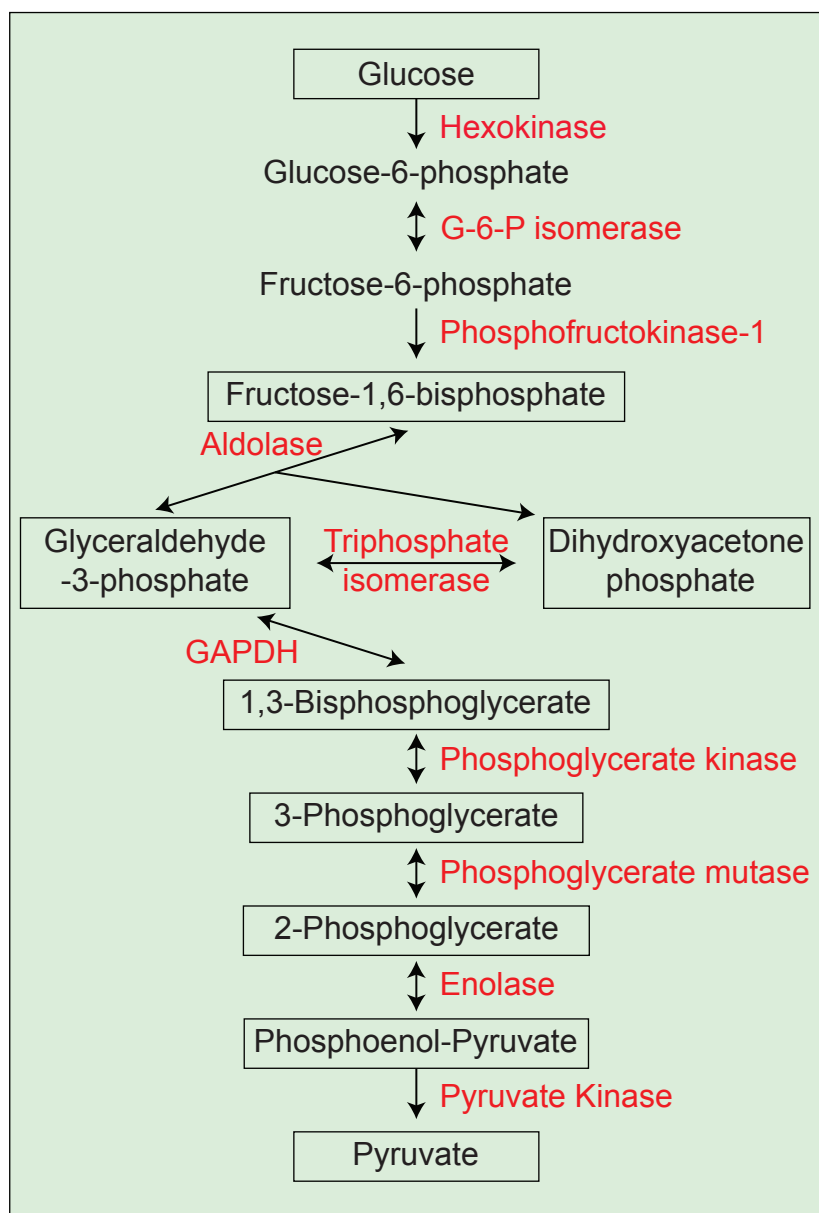

B

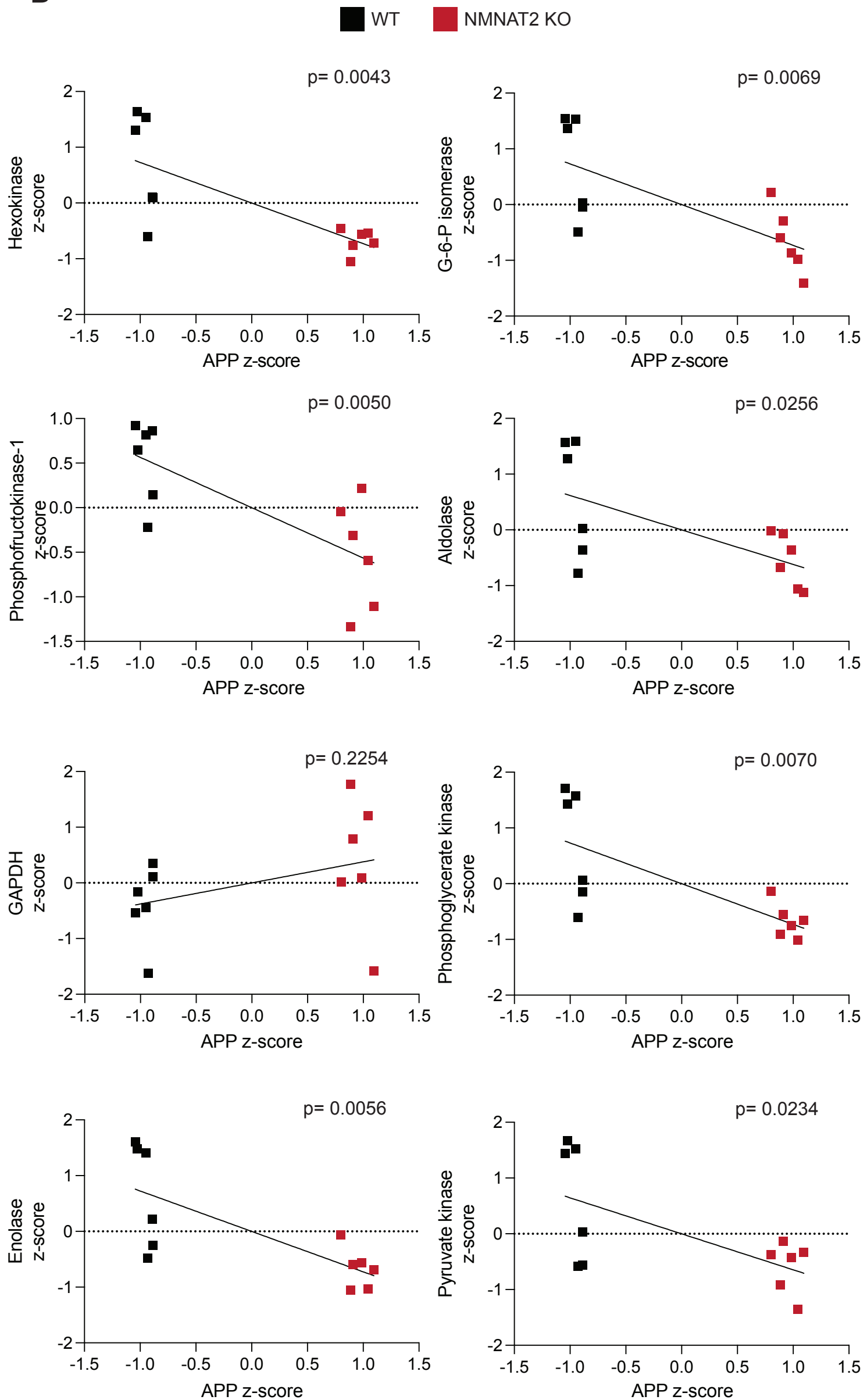

Supplement: Supplementary file 1 [file cells-15-01100-s001.zip › Supp Figure S2.pdf]

■ WT ■ NMNAT2 KO

A

Complex I

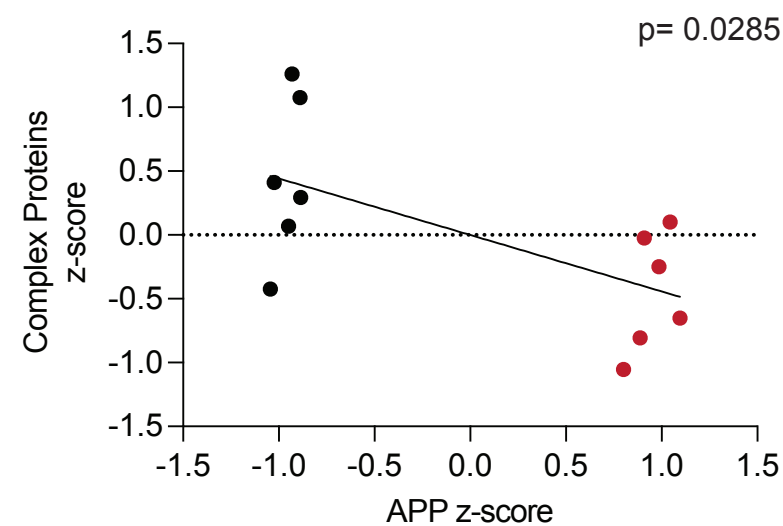

B

Complex II

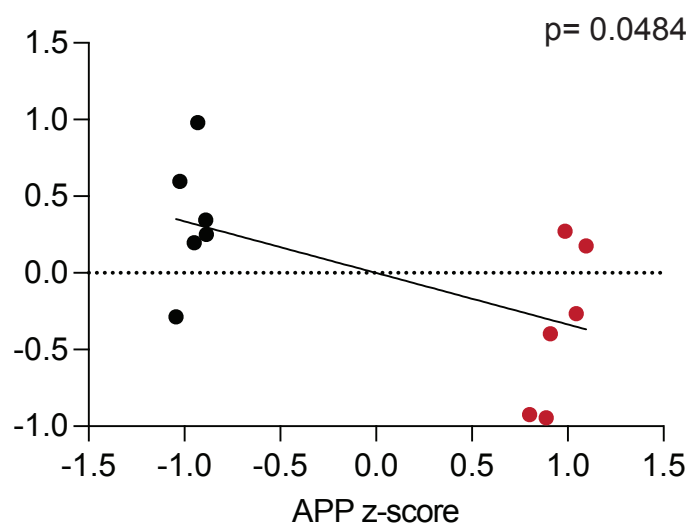

C

Complex III

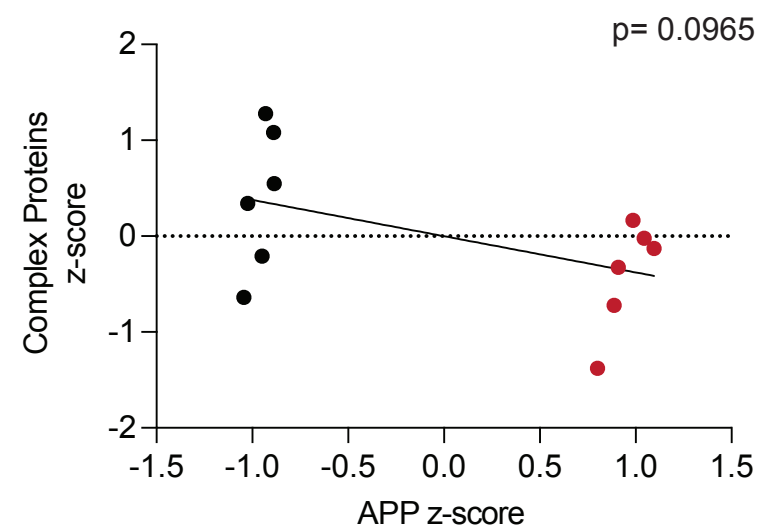

D

Complex IV

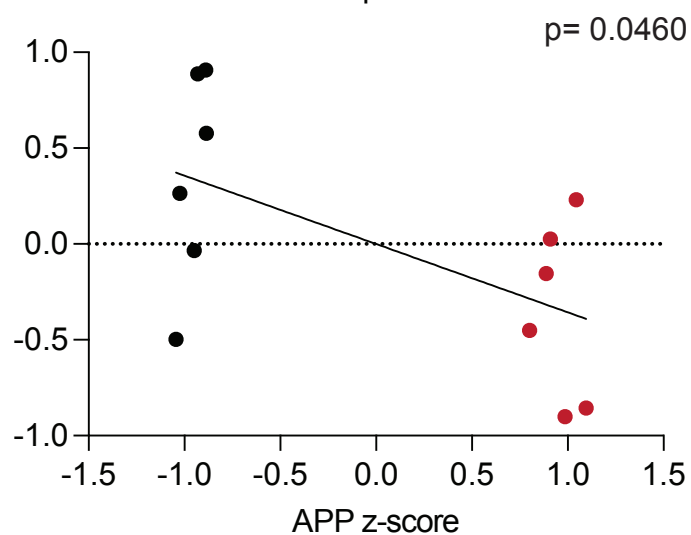

E

Complex V

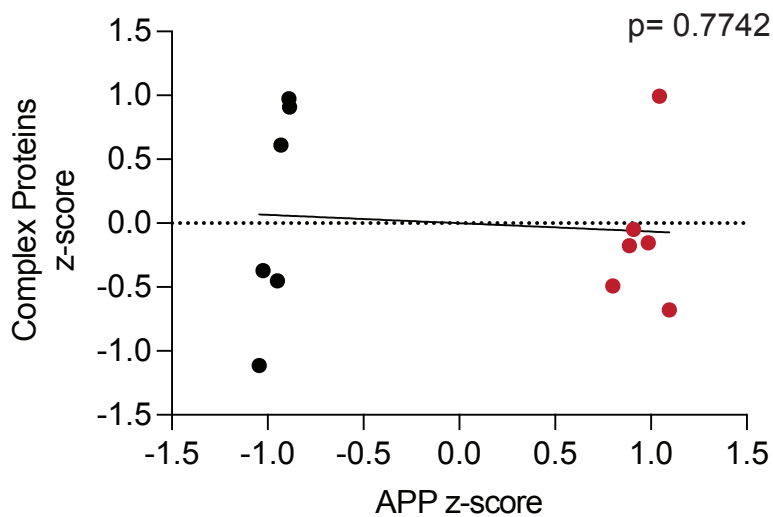

Supplement: Supplementary file 1 [file cells-15-01100-s001.zip › Supp Figure S3.pdf]
